# Deletion or inhibition of soluble epoxide hydrolase protects against brain damage and reduces microglia-mediated neuroinflammation in traumatic brain injury

**DOI:** 10.18632/oncotarget.21139

**Published:** 2017-09-21

**Authors:** Tai-Ho Hung, Song-Kun Shyue, Chun-Hu Wu, Chien-Cheng Chen, Chao-Chang Lin, Che-Feng Chang, Szu-Fu Chen

**Affiliations:** ^1^ Department of Obstetrics and Gynecology, Chang Gung Memorial Hospital at Taipei and College of Medicine, Chang Gung University, Taoyuan, Taiwan, Republic of China; ^2^ Institute of Biomedical Sciences, Academia Sinica, Taipei, Taiwan, Republic of China; ^3^ Graduate Institute of Life Sciences, National Defense Medical Center, Taipei, Taiwan, Republic of China; ^4^ Department of Physical Medicine and Rehabilitation, Cheng Hsin General Hospital, Taipei, Taiwan, Republic of China; ^5^ Department of Neurology, Yale University School of Medicine, New Haven, Connecticut, USA; ^6^ Departments of Physiology and Biophysics, National Defense Medical Center, Taipei, Taiwan, Republic of China

**Keywords:** soluble epoxide hydrolase, traumatic brain injury, microglia, inflammation, AUDA

## Abstract

Traumatic brain injury (TBI) induces a series of inflammatory processes that contribute to neuronal damage. The present study investigated the involvement of soluble epoxide hydrolase (sEH) in neuroinflammation and brain damage in mouse TBI and in microglial cultures. The effects of genetic deletion of sEH and treatment with an sEH inhibitor, 12-(3-adamantan-1-yl-ureido)-dodecanoic acid (AUDA), on brain damage and inflammatory responses were evaluated in mice subjected to controlled cortical impact. The anti-inflammatory mechanism of sEH inhibition/deletion was investigated *in vitro*. TBI-induced an increase in sEH protein level in the injured cortex from 1 h to 4 days and sEH was expressed in microglia. Genetic deletion of sEH significantly attenuated functional deficits and brain damage up to 28 days post-TBI. Deletion of sEH also reduced neuronal death, apoptosis, brain edema, and BBB permeability at 1 and 4 day(s). These changes were associated with markedly reduced microglial/macrophage activation, neutrophil infiltration, matrix metalloproteinase-9 activity, inflammatory mediator expression at 1 and 4 day(s), and epoxyeicosatrienoic acid (EET) degradation at 1 and 4 day(s). Administration of AUDA attenuated brain edema, apoptosis, inflammatory mediator upregulation and EET degradation at 4 days. In primary microglial cultures, AUDA attenuated both LPS- or IFN-γ-stimulated nitric oxide (NO) production and reduced LPS- or IFN-γ-induced p38 MAPK and NF-κB signaling. Deletion of sEH also reduced IFN-γ-induced NO production. Moreover, AUDA attenuated N2A neuronal death induced by BV2 microglial-conditioned media. Our results suggest that inhibition of sEH may be a potential therapy for TBI by modulating the cytotoxic functions of microglia.

## INTRODUCTION

Traumatic brain injury (TBI) is the leading cause of mortality and long-term disability among young adults worldwide [1]. TBI induces brain damage due to initial physical disruption of tissue (primary injury) and subsequent development of excitotoxicity, oxidative damage, and inflammation (secondary injury) [2]. Of these, cerebral inflammation is regarded as a key factor in the secondary injury cascade and contributes to neuronal death and neurological deterioration [3, 4]. Post-traumatic inflammatory responses are mediated by activation of microglia and recruitment of peripheral leukocytes to the cerebral parenchyma [4]. Activated microglia produce multiple proinflammatory mediators, including cytokines, chemokines, nitric oxide (NO) and other factors with cytotoxic effects. Overproduction of these mediators contributes to neuronal damage and blood-brain barrier (BBB) disruption, which is considered to be the major cause of vasogenic brain edema and subsequent brain injury [3–5]. Microglia can be further activated by various signals released from dying neurons or by direct contact with damaged neurons, thus further amplifying neuronal damage [4, 6]. Activated microglia can induce mitochondria-dependent apoptosis via production of reactive oxygen and nitrogen species [6]. Additionally, proinflammatory cytokines derived from microglia can activate receptor-dependent apoptotic pathways via recruitment of adaptor molecules and caspase-8 or -10 activation [7, 8]. Accordingly, inhibition of microglial activation and, therefore, production of inflammatory mediators has been suggested to be a potential therapeutic strategy for protecting the damaged brain in TBI [9, 10].

Epoxyeicosatrienoic acids (EETs), which are produced from arachidonic acid in a cytochrome P450-catalyzed reaction, have potent anti-inflammatory actions [11]. Previous studies have shown that exogenous administration of 14,15- EET and increased expression of CYP epoxygenases in the brain are protective against experimental ischemic brain injury [12, 13]. *In vitro* and *in vivo* studies have also shown that EETs possess potent anti-inflammatory effects [11, 12, 14, 15], suggesting that EETs signaling may suppress trauma-evoked neuroinflammation. EETs are broken down into less bioactive corresponding dihydroxyeicosatrienoic acids (DHETs) by soluble epoxide hydrolase enzyme (sEH), and inhibition of sEH increases EETs levels in tissues and plasma, thus enhancing the effects of EETs [11]. The significance of sEH in damaged brain is supported by evidence that the expression of this enzyme is significantly increased in rodent models of brain damage including cerebral ischemia [12], Parkinsonism [16], and seizures [17]. Clinically, patients with genetic polymorphisms that reduce sEH activity have improved outcomes after subarachnoid hemorrhage [18]. Genetic deletion or pharmacological inhibition of the epoxide hydrolase gene has also been shown to provide protection against experimental cerebral ischemia [19, 20], Parkinsonism [16] and seizure [17].

Although genetic deletion of sEH has been reported to improve functional outcomes after mouse TBI [21], little is known about the role of sEH in regulating microglia-induced neuroinflammation in brain damage. It is also unclear whether sEH can be activated by TBI or whether sEH inhibition has an impact on neuronal damage and brain edema. To address these issues, the present study aimed to examine the effects of pharmacological and genetic inhibition of sEH on long-term functional impairment, neuronal damage, and brain edema after experimental TBI in mice. We further aimed to investigate whether sEH inhibition attenuated TBI-induced microglial activation, thereby preventing neuronal damage in both cell and animal models.

## RESULTS

### Increased sEH expression in mice after TBI

**Figure 1:**
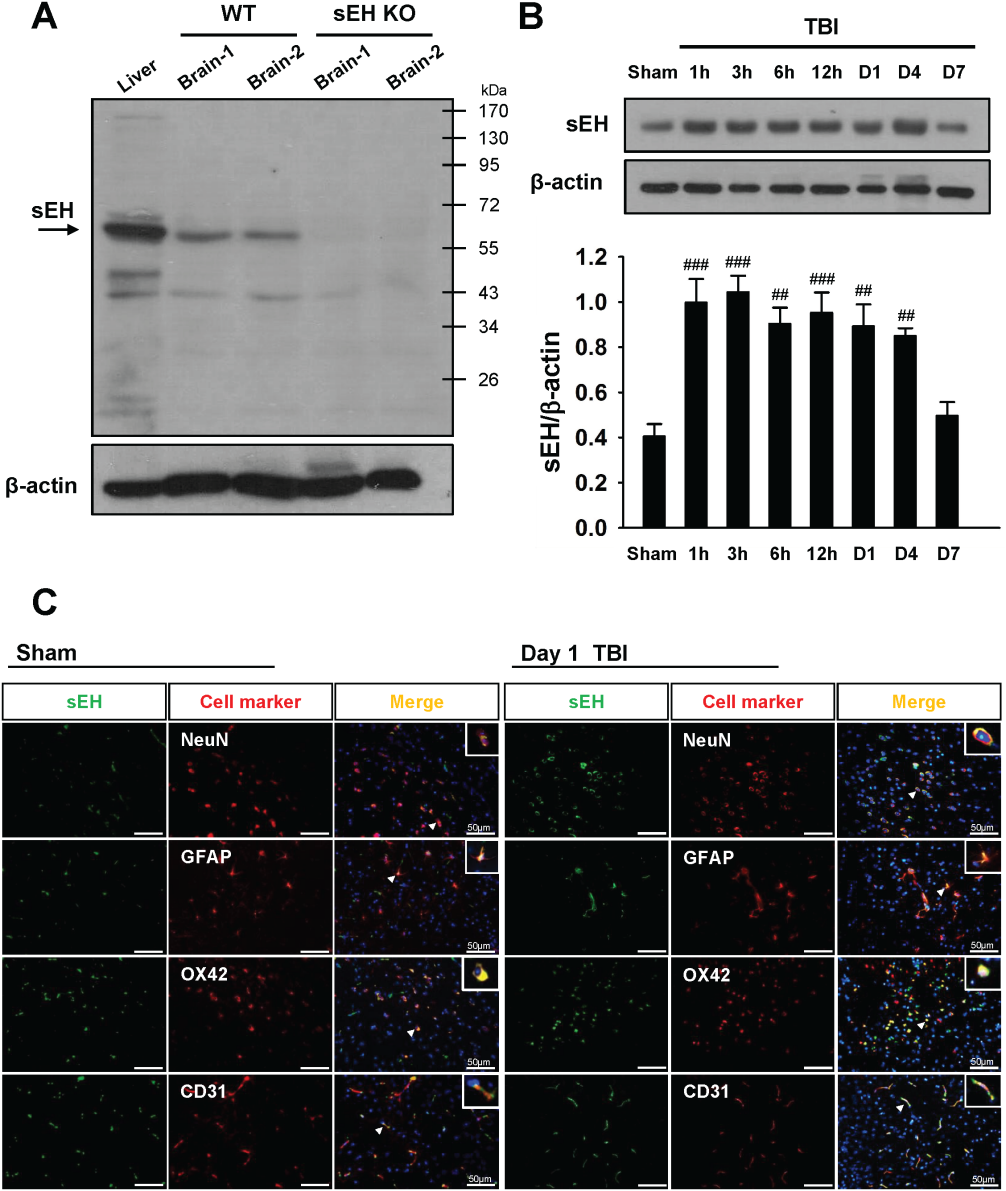
Upregulation of sEH protein expression in WT brains subjected to TBI **(A)** Western blot analysis of mouse liver and brain tissues using anti-sEH antibody demonstrating a dominant band at ∼62 kDa in the WT liver (lane 1) and WT brains (lanes 2 and 3), whereas no product was present in the sEH KO brains (lanes 4 and 5), confirming the specificity of the antibody. **(B)** Representative immunoblots of the sEH protein in the ipsilateral hemispheres of mice subjected to CCI injury or sham surgery. Bar graph of densitometric analysis of bands showing a significant increase in sEH protein level in the ipsilateral hemispheres of WT mice at 1 h, 3 h, 6 h, 12 h, 1 day and 4 days post-TBI, compared with the sham-operated brains. At 7 days after injury, the sEH protein level had decreased to the sham level. **(C)** Identification of sEH -positive cells at 1 day post-TBI or sham surgery in the peri-contussional area by double immunofluorescence labeling. sEH immunoreactivity is shown in green, and immunolabeling of NeuN (neurons), GFAP (astrocytes), OX42 (microglia), or CD31 (endothelial cells) is shown in red. Co-localization is shown by yellow labeling. Arrowheads indicate the location of higher magnification images. sEH localized to neurons, astrocytes, microglia, and endothelial cells. Sections were stained with DAPI (blue) to show all nuclei. Values are mean ± S.E.M; ^##^*P* < 0.01, and ^###^*P* < 0.001 vs. sham group (n = 5–6 mice / group, one-way ANOVA). The scale bar is 50 μm.

We first verified the specificity of the sEH antibody by immunoblotting on the wild-type (WT) liver tissue (the positive control) and brain samples of sEH knockout (KO) mice (the negative control). Western blot analysis of liver and brain homogenates from WT mice revealed the presence of a dominant sEH-immunoreactive band at ∼62 kDa, which was absent in the brain of sEH KO mice (Figure 1A), confirming the specificity of the antibody to sEH.

To investigate the possible participation of sEH in the pathogenesis of TBI, we first examined the expression and cellular localization of sEH in brains subjected to controlled cortical impact (CCI) injury. Compared with the sham control group, CCI induced an increase in sEH protein level in the injured cortex at 1 h, 3 h, 6 h, 12 h, 1 day and 4 days (all *P* < 0.01; Figure 1B). At 7 days after injury, the sEH protein level had decreased to the sham level. We also investigated the cellular localizations of sEH at 1 day post-injury or following sham surgery. Dual-label immunofluorescence demonstrated that sEH was expressed in microglia, neurons, astrocytes, and endothelial cells in the contusion margin of the injury core or sham-operated brains (Figure 1C). No immunoreactivity was observed in either hemisphere of sEH KO mice after CCI (Supplementary Figure 1).

### Deletion of sEH reduces long-term neurological deficits and brain tissue damage after TBI

**Figure 2:**
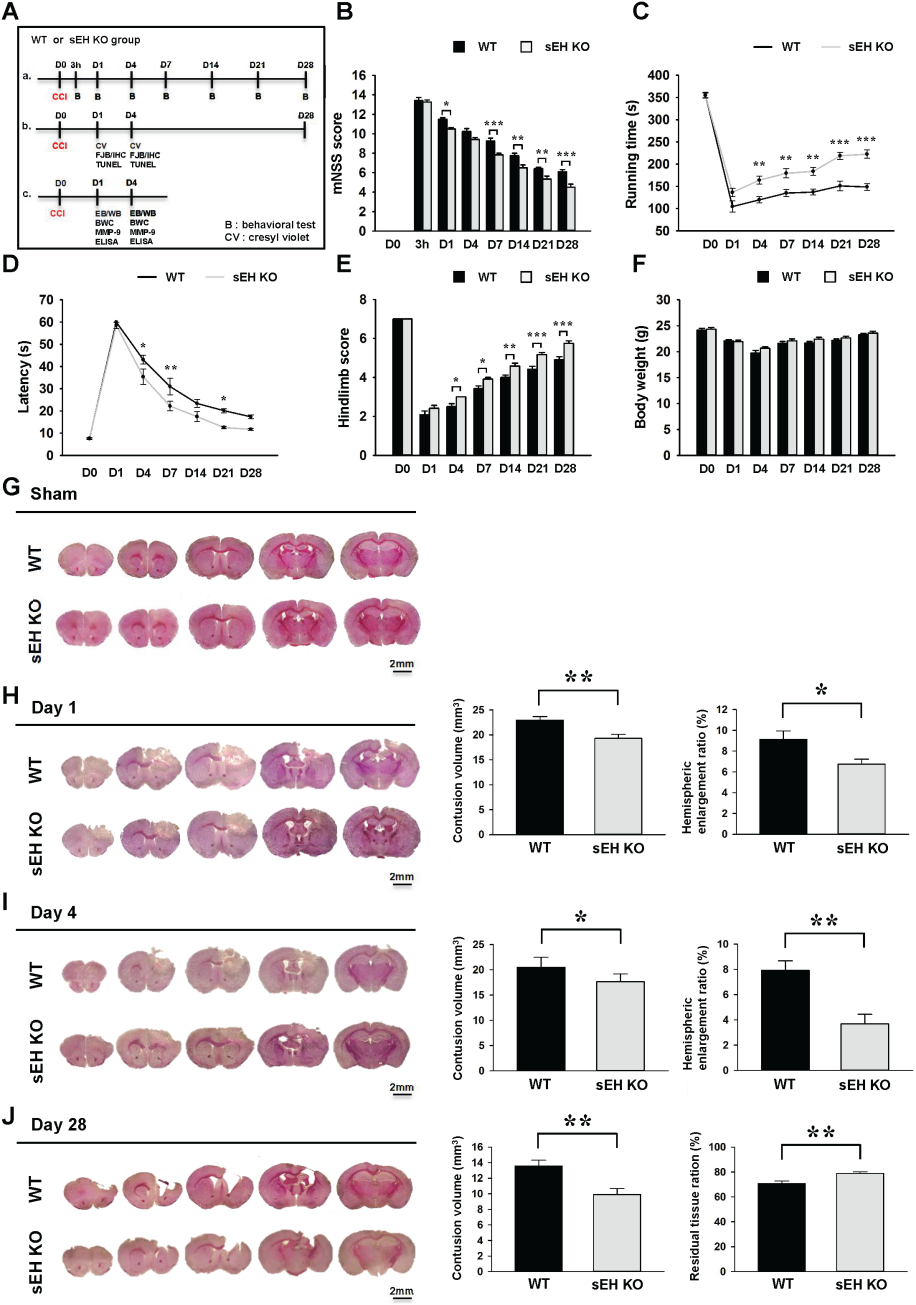
Deletion of sEH improved long-term neurobehavioral functions and reduced brain tissue damage after TBI **(A)** Experimental design and animal groups classification. TBI: traumatic brain injury; B: behavioral test; CV: cresyl violet; IF: immunofluorescence staining; FJB: fluoro-jade Bs staining; IHC: immunohistochemical staining; TUNEL: terminal deoxynucleotidyl transferase-mediated dUTP-nick end labeling; EB: Evans Blue; WB: Western blots; BWC: brain water content; MMP-9: matrix metalloproteinase-9; ELISA: enzyme-linked immunosorbent assay. Deletion of sEH (compared with WT control) significantly **(B)** reduced the modified neurological severity score (mNSS) at 1, 7, 14, 21 and 28 days post-injury, **(C)** improved the rotarod performance from 4 to 28 days post-injury, **(D)** reduced the beam walk traversing time at 4, 7 and 21 days, and **(E)** improved hindlimb function from 4 to 28 days. **(F)** There were no significant differences in body weight change between sEH KO and WT mice during the 28-day observation period post-TBI. Representative cresyl violet-stained brain sections of WT and sEH KO mice **(G)** following sham surgery, and at **(H)** 1 day, **(I)** 4 days and **(J)** 28 days post-TBI. Analysis of lesion volumes demonstrated that deletion of sEH significantly reduced contusion volume and hemispheric enlargement at both 1 and 4 days, and significantly reduced contusion volume and preserved brain tissue at 28 days. The scale bar is 2 mm. Values are mean ± S.E.M; *P < 0.05, **P < 0.01 and ***P < 0.001vs. WT group (n = 12 mice / group for behavior tests, repeated measures two-way ANOVA; n = 7 mice / group for histology, Student’s *t*-test).

To assess whether sEH contributes to TBI, we first used a loss-of-function strategy to evaluate the effect of sEH deletion on behavioral recovery (Figures 2A-2E). The modified neurological severity score (mNSS) was used to measure global neurological deficits. At 3 h after injury, there was no difference in mNSS between WT and sEH KO mice, indicating that injury severity was initially equal (Figure 2B). The mNSS scores were significantly lower for sEH KO mice than for WT mice at 1, 7, 14, 21, and 28 days (all *P* < 0.05; Figure 2B). Rotarod and beam walking tests were employed to assess motor and coordination functions. Rotarod performance was significantly better in sEH KO mice compared with WT mice from 4 to 28 days (all *p* < 0.01; Figure 2C). In the beam-walking test, sEH KO mice required significantly less time to cross the beam at 4, 7 and 21 days post-injury (all *P* < 0.05; Figure 2D). The hindlimb scores were also significantly higher for sEH KO than for WT mice from 4 to 28 days (all *P* < 0.05; Figure 2E). However, there were no significant changes between the two groups in body weight (Figure 2F). Overall, these findings suggest that sEH deletion improves long-term neurobehavioral outcomes following CCI.

To test whether genetic deletion of sEH also has an impact on brain tissue damage, we next analyzed the contusion volume and the ipsilateral hemispheric enlargement, an indicator of brain swelling, at the acute phase of TBI. Deletion of the sEH gene significantly attenuated contusion volume to 83.9% of the WT level from 23.0 ± 0.7 mm^3^ to 19.3 ± 0.8 mm^3^ at day 1 (*P* = 0.0051; Figure 2H), and to 85.9% of the WT level from 20.5 ± 0.7 mm^3^ to 17.6 ± 0.6 mm^3^ at day 4 (*P* = 0.01; Figure 2I). Similarly, sEH KO mice also displayed 26.4% less ipsilateral hemispheric enlargement at day 1 (6.7 ± 0.5% vs. 9.1 ± 0.8%, *P* = 0.0298; Figure 2H) and 53.2% less at day 4 (3.7 ± 0.8% vs. 7.9 ± 0.8%, *P* = 0.002; Figure 2I). We next analyzed brain tissue damage and the remaining ipsilateral hemisphere volume during the chronic phase of TBI. Consistent with the results at 1 day and 4 days, CCI induced a pronounced tissue loss in the injured hemisphere at post-injury day 28. The contusion volume in sEH KO mouse brains was only 72.8% of that in WT mouse brains (9.9 ± 0.8 mm^3^ vs. 13.6 ± 0.7 mm^3^, *P* = 0.0046; Figure 2J) at day 28. Likewise, there was greater preservation of brain tissue in sEH KO mice (78.9 ± 1.3% of the contralateral hemisphere) compared with WT mice (70.7 ± 2.1%, *P* = 0.006; Figure 2J).

### Deletion of sEH reduces neuronal damage and apoptosis after TBI

**Figure 3:**
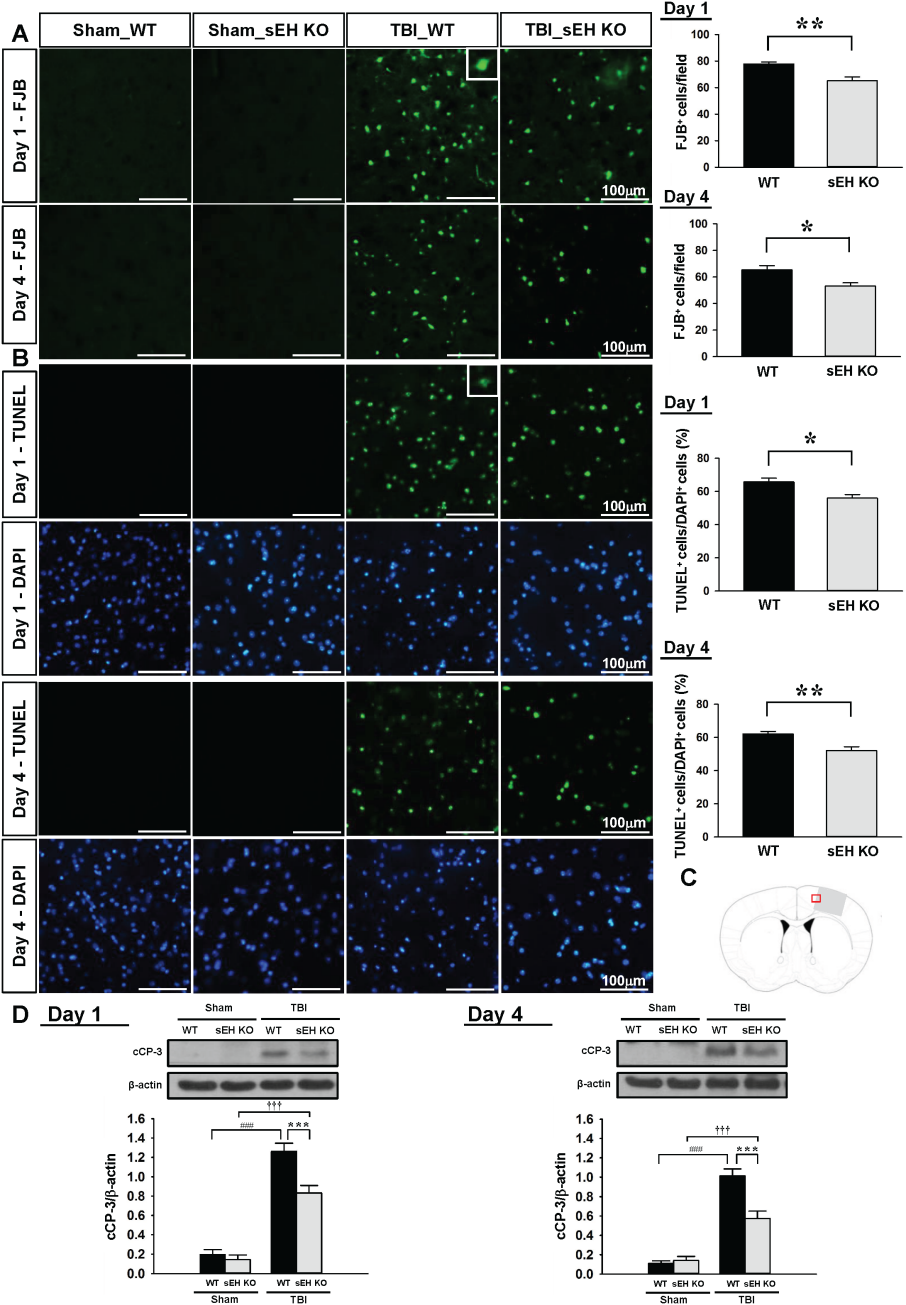
Deletion of sEH reduced neuronal damage and apoptotic cell death in mice after TBI **(A)** Representative FJB, and **(B)** TUNEL (green) and DAPI-stained (blue) brain sections of WT and sEH KO mice at 1 and 4 days post-TBI. The inset is a representative FJB-positive or TUNEL-positive cell at higher magnification. Quantification analysis indicates that sEH KO mice had significantly fewer degenerating neurons and a lower percentage of TUNEL-positive cells than WT mice at both 1 and 4 days post-TBI. The total number of FJB-positive cells is expressed as the mean number per field of view (0.8 mm^2^). The percentage of TUNEL-positive cells is expressed as the percentage of nuclei that were stained by the TUNEL method/the total number of DAPI-stained nuclei. Sections were stained with DAPI (blue) to show all nuclei. **(C)** Brain atlas coronal brain section of a core injury region at 0.74 mm from the bregma. The red box indicates the the location of representative images. The scale bar is 100 μm. **(D)** Western blot analysis of cleaved caspase-3 in the ipsilateral hemisphere of WT and sEH KO mice at 1 and 4 days following sham surgery or TBI. Deletion ofsEH significantly decreased the cleaved caspase-3 level at both 1 and 4 days post-TBI. cCP-3: cleaved caspase-3. Values are mean ± S.E.M; ^###^, ^†††^ *P* < 0.001 vs. sham group; *P < 0.05, **P < 0.01, ***P < 0.001 vs. WT group (n = 7 mice / group, Student’s *t*-test for histology and repeated measures two-way ANOVA for Western blots).

We further investigated whether deletion of sEH influenced neuronal damage and cell apoptosis at 1 day and 4 days post-injury. We chose these 2 time-points because previous studies have shown that Fluoro-Jade B and (FJB) and terminal deoxynucleotidyl transferase-mediated dUTP-biotin nick end labeling (TUNEL) reactivity peak at 1 day after experimental TBI, last for over 3 days and are almost absent after 14 days [22, 23]. In line with the results of cresyl violet staining, the number of FJB-positive neurons in the contusion margin of the injury core was significantly reduced in sEH KO mice compared with WT mice at both 1 day (65.2 ± 2.8 vs. 77.8 ± 1.5 cells/field, *P* = 0.002; Figure 3A) and 4 days (53.2 ± 2.5 vs. 65.3 ± 3.3 cells/field, *P* = 0.0117; Figure 3A). Additionally, deletion of sEH significantly diminished the number of TUNEL-positive cells in the contusion margin at both 1 day (55.9 ± 2.2% vs. 65.7 ± 2.3%, *P* = 0.0105; Figure 3B) and 4 days (52.1 ± 2.3% vs. 62.2 ± 1.5%, *P* = 0.0034; Figure 3B). The level of cleaved caspase-3, a final effector of apoptotic death, was significantly elevated at both tested time-points following CCI in both WT and sEH KO groups (all *P* < 0.001; Figure 3D). The cleaved caspase-3 level in sEH KO mouse injured brains was significantly decreased to 65.9% (*P* < 0.001) of the WT-level at 1 day and to 56.7% (*P* < 0.001) of the WT-level at 4 days (Figure 3D). Taken together, these results demonstrate that deletion of sEH provides neuroprotection following TBI.

**Figure 4:**
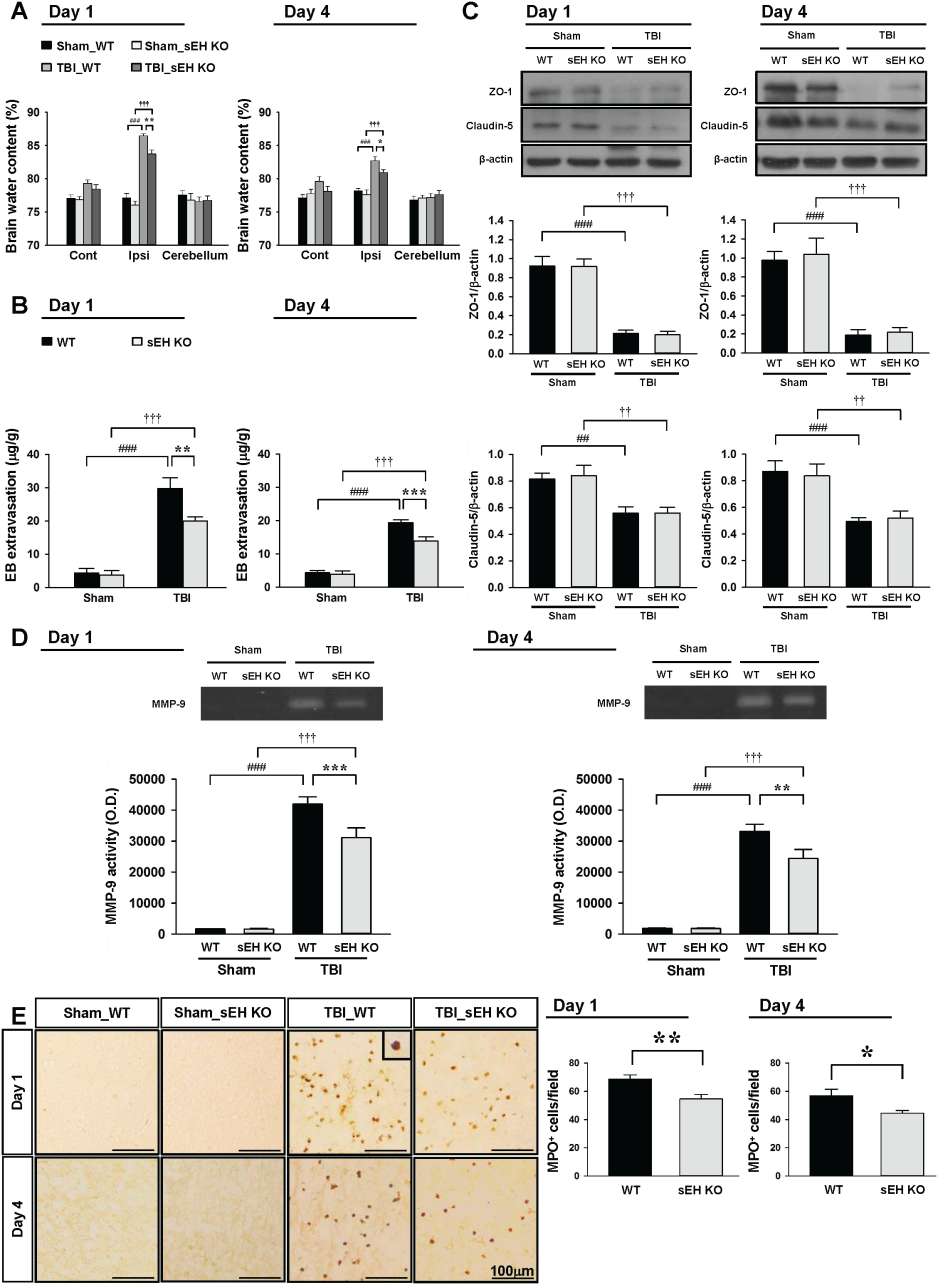
Deletion of sEH attenuates brain edema, BBB permeability, MMP-9 enzymatic activity and neutrophil infiltration after TBI Deletion of sEH significantly decreased **(A)** brain water content and **(B)** leakage of Evans blue into the brain and in the ipsilateral hemisphere compared with the WT mice. Cont: contralateral cortex; Ipsi: ipsilateral cortex. **(C)** Western blot analysis of ZO-1 and claudin-5 in the ipsilateral hemisphere of WT and sEH KO mice at 1 and 4 days following sham surgery or TBI. Deletion of sEH did not affect TBI-mediated reduced expression of ZO-1 or claudin-5 at any tested time-points. **(D)** Representative zymography of MMP-9 activity from WT and sEH KO mice at 1 and 4 days following sham surgery or TBI. The gelatinase activity of MMP-9 was significantly decreased in sEH KO mice compared with WT control at both 1 and 4 days post-TBI. **(E)** Representative MPO-stained brain images of WT and sEH KO mice in the contusion margin (see the red box in the brain atlas coronal brain section in Figure 3C) at 1 day and 4 days following sham surgery or TBI. The inset is a representative MPO-positive cell at higher magnification. Cell count analysis shows that sEH KO mice had significantly fewer infiltrating neutrophils than WT control in the cortical contusion margin at 1 and 4 days post-TBI. The number of MPO-positive cells is expressed as the mean number per field of view (0.8 mm^2^). The scale bar is 100 μm. Values are mean ± S.E.M; ^##^, ^††^ *P* < 0.01, ^###^, ^†††^ *P* < 0.001 vs. sham group; *P < 0.05, **P < 0.01, ***P < 0.001 vs. WT group (n = 6–7 mice / group for brain water content and Evans blue amount, and n = 6 mice / group for Western blot analysis and MMP-9 activity, repeated measures two-way ANOVA; n = 6 mice / group for MPO staining, Student’s *t*-test).

### Deletion of sEH attenuates brain edema, BBB permeability, MMP-9 enzymatic activity and neutrophil infiltration after TBI

To investigate the potential mechanism of sEH in regulating TBI-triggered inflammation, we first measured brain edema and BBB breakdown following CCI as both are a consequence of post-injury inflammation [24]. In both WT and sEH KO mice, brain water content in the ipsilateral hemisphere of the TBI groups was significantly higher than that in the sham groups at both 1 and 4 days (all *P* < 0.001; Figure 4A). Compared with WT mice, sEH KO mice had significantly decreased brain water content in the ipsilateral hemisphere at both 1 (83.7 ± 0.6% vs. 86.5 ± 0.3%, *P* = 0.002; Figure 4A) and 4 days (81.0 ± 0.4% vs. 82.7 ± 0.6%, *P* = 0.029; Figure 4A). As BBB breakdown may result in the accumulation of circulating fluid and cause brain edema [25], we further evaluated whether deletion of sEH attenuated BBB breakdown, as determined by leakage of albumin-bound Evans blue into the brain. In both WT and sEH KO mice, there was a marked increase in Evans blue extravasation in the ipsilateral hemisphere of the TBI groups compared with the sham groups at both 1 and 4 days (all *p* < 0.001; Figure 4B). The Evans blue content in the ipsilateral hemisphere of the WT groups was significantly attenuated by sEH deletion at both 1 day (20.0 ± 1.2 μg/g vs. 29.8 ± 3.2 μg/g; *P* = 0.002; Figure 4B) and 4 days (13.9 ± 1.3 μg/g vs. 19.4 ± 0.9 μg/g; *p* < 0.001; Figure 4B) post-TBI.

We also examined the effects of sEH deletion on two major proteins involved in the tight junctions of the BBB, zonula occludens (ZO)-1 and claudin-5. In both WT and sEH KO mice, TBI resulted in a significant decrease in both ZO-1 and caludin-5 protein expression at 1 and 4 days after injury (all *p* < 0.01; Figure 4C). However, there was no significant difference in ZO-1 nor claudin-5 expression between the two groups at either tested time-point. We next investigated matrix metalloproteinase (MMP)-9 activity and the degree of neutrophil infiltration, both of which contribute to BBB breakdown and brain edema [24]. MMP-9 is an endopeptidase and functions to degrade the extracellular matrix, including major components of the endothelial basal lamina, which is essential in maintaining BBB integrity [24]. MMP-9 activity was significantly increased in both WT mouse and sEH KO mouse injured brains at 1 and 4 days post-TBI (all *p* < 0.001; Figure 4D). In sEH KO mice, MMP-9 activity was significantly decreased compared with WT mice at both tested time-points (both *p* < 0.01; Figure 4D). Moreover, TBI induced robust infiltration of neutrophils in both WT and sEH KO mice (Figure 4E). There were significantly fewer neutrophils in the in the contusion margin of sEH KO mouse brains compared with WT mouse brains at both 1 day (54.6 ± 3.1 vs. 68.7 ± 2.9 cells/field, *P* = 0.0082; Figure 4E) and 4 days (44.4 ± 2.0 vs. 56.8 ± 4.6 cells/field, *P* = 0.0347; Figure 4E) post-TBI.

### Deletion of sEH reduces proinflammatory M1 microglia/macrophage activation and heightens anti-inflammatory M2 microglia/macrophage response, and attenuates EET degradation after TBI

**Figure 5:**
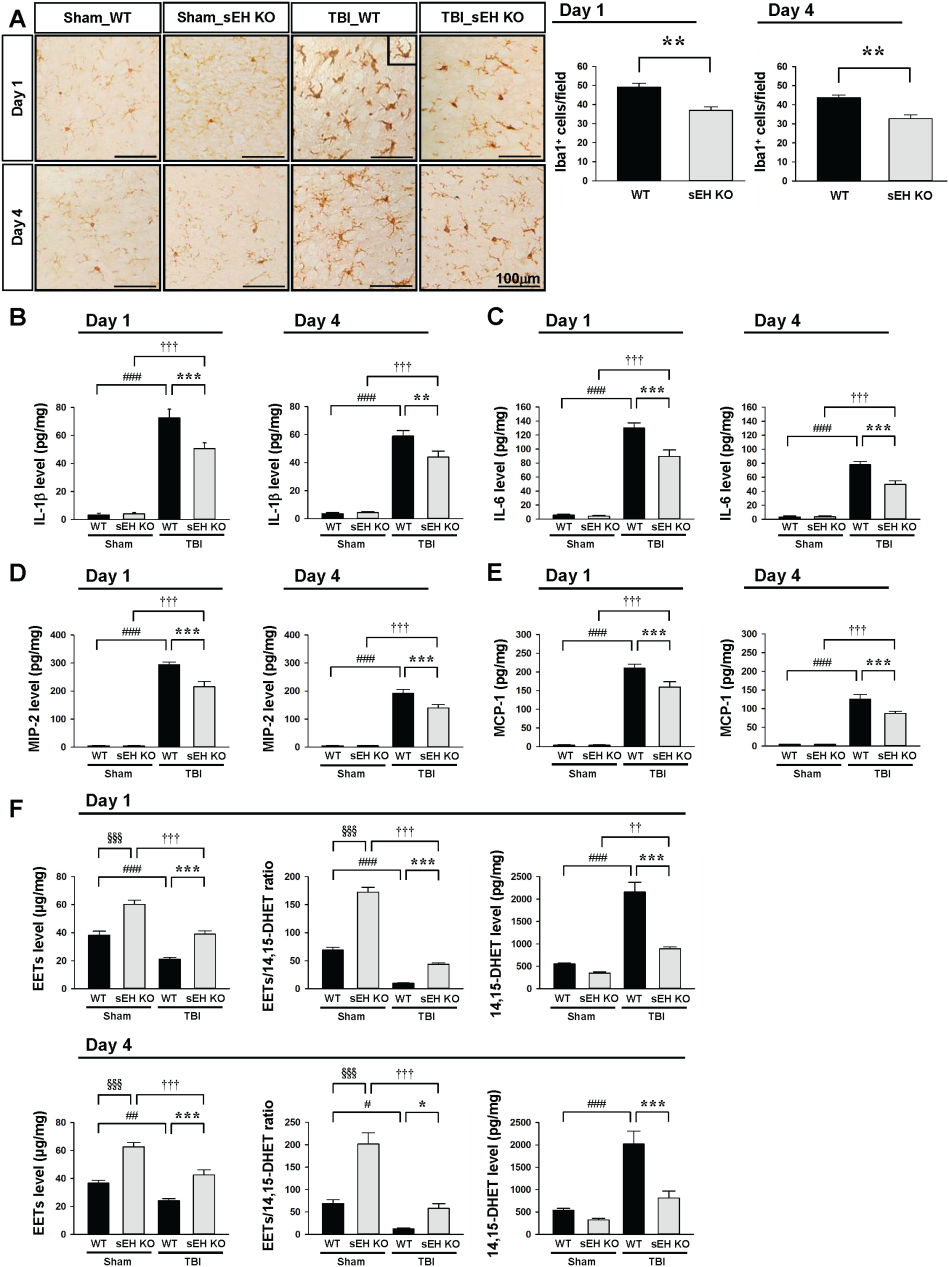
Deletion of sEH reduced microglial/macrophage activation and expression of inflammatory cytokines and chemokines and EET degradation Representative **(A)** Iba1-stained images from WT and sEH KO mice in the contusion margin (see the red box in the brain atlas coronal brain section in Figure 3C) at 1 day and 4 days following sham surgery or TBI. Cell count analysis shows that sEH KO mice had significantly fewer Iba1-positive cells than WT in the cortical contusion margin at both 1 and 4 days post-TBI. The number of Iba1-positive cells is expressed as the mean number per field of view (0.8 mm^2^). The scale bar is 100 μm. Bar graphs demonstrating **(B)** IL-1β, **(C)** IL-6, **(D)** MIP-2, **(E)** MCP-1, **(F)** EET level, EET/14,15 DHET ratio and 14,15 DHET protein level, as assessed by ELISA in the ipsilateral cortices of WT and sEH KO mice at 1 day or 4 days following sham surgery or TBI. Deletion of sEH significantly attenuated IL-1β, IL-6, MIP-2, and MCP-1 protein levels compared with WT mice at 1 day and 4 days post-TBI. sEH KO mice exhibited significantly increased EETs level, elevated EET/ 14,15 DHET ratio and reduced 14,15 DHET level compared with WT mice at both 1 day and 4 days post-TBI. Values are mean ± S.E.M; ^†^ *P* < 0.05, ^##^ *P* < 0.01, ^###^, ^†††^ *P* < 0.001 vs. sham group; *P < 0.05, ^***, §§§^*P* < 0.001 vs. WT group (n = 6–7 mice / group for cytokines and chemokines, and n = 4–5 mice / group for EETs and 14, 15 DHET, repeated measures two-way ANOVA).

We further assessed whether sEH deletion affected microglial activation and the expression of inflammatory mediators, both of which are key inflammatory processes following TBI. Activated microglia/macrophages were observed within the injured cortex at both 1 and 4 days, and the number of activated microglia/macrophages was significantly reduced in sEH mice compared with WT mice at 1 day (36.9 ± 1.9 vs. 49.1 ± 2.0 cells/field, *P* = 0.0014; Figure 5A) and 4 days (32.8 ± 2.0 vs. 43.7 ± 2.4 cells/field, *P* = 0.001; Figure 5A). With regard to inflammatory mediators, TBI induced increases in IL-1β, IL-6, MIP-2, and MCP-1 protein expression levels in both WT and sEH KO mice at 1 and 4 days (Figures 5B-5E). The injured hemispheres of sEH KO mice exhibited significantly reduced IL-1β, IL-6, MIP-2, and MCP-1 protein levels compared with WT mice at 1 day (IL-1β: 50.6 ± 4.2 vs. 72.7 ± 6.1 pg/mg protein, *P* < 0.001; Figure 5B; IL-6: 89.6 ± 9.2 vs. 130.0 ± 7.4 pg/mg protein, *P* < 0.001; Figure 5C; MIP-2: 215.7 ± 18.3 vs. 294.0 ± 9.5 pg/mg protein, *P* < 0.001; Figure 5D; MCP-1: 159.6 ± 14.2 vs. 210.1 ± 10.4 pg/mg protein, *P* < 0.001; Figure 5E) and 4 days (IL-1β: 44.0 ± 4.3 vs. 59.0 ± 4.0 pg/mg protein, *P* = 0.002; Figure 5B; IL-6: 49.8 ± 5.0 vs. 78.0 ± 4.5 pg/mg protein, *P* < 0.001; Figure 5C; MIP-2: 140.0 ± 11.6 vs. 191.9 ± 13.9 pg/mg protein, *P* < 0.001; Figure 5D; MCP-1: 87.3 ± 5.9 vs. 125.8 ± 12.3 pg/mg protein, *P* < 0.001; Figure 5E). In addition, TBI induced reductions in EET level and EET/14,15-DHET ratio, and an increase in 14,15-DHET level in WT mice at both 1 day and 4 days post-injury (all *P* < 0.05; Figures 5F). Deletion of sEH gene caused a significant elevation of EET level (*P* < 0.001 for both 1 day and 4 days) and EET/14,15-DHET ratio (*P* < 0.001 for 1 day and *P* = 0.042 for 4 days), and a decrease of 14,15-DHET level (*P* < 0.001 for both 1 day and 4 days; Figure 5G) at both tested time-points.

**Figure 6:**
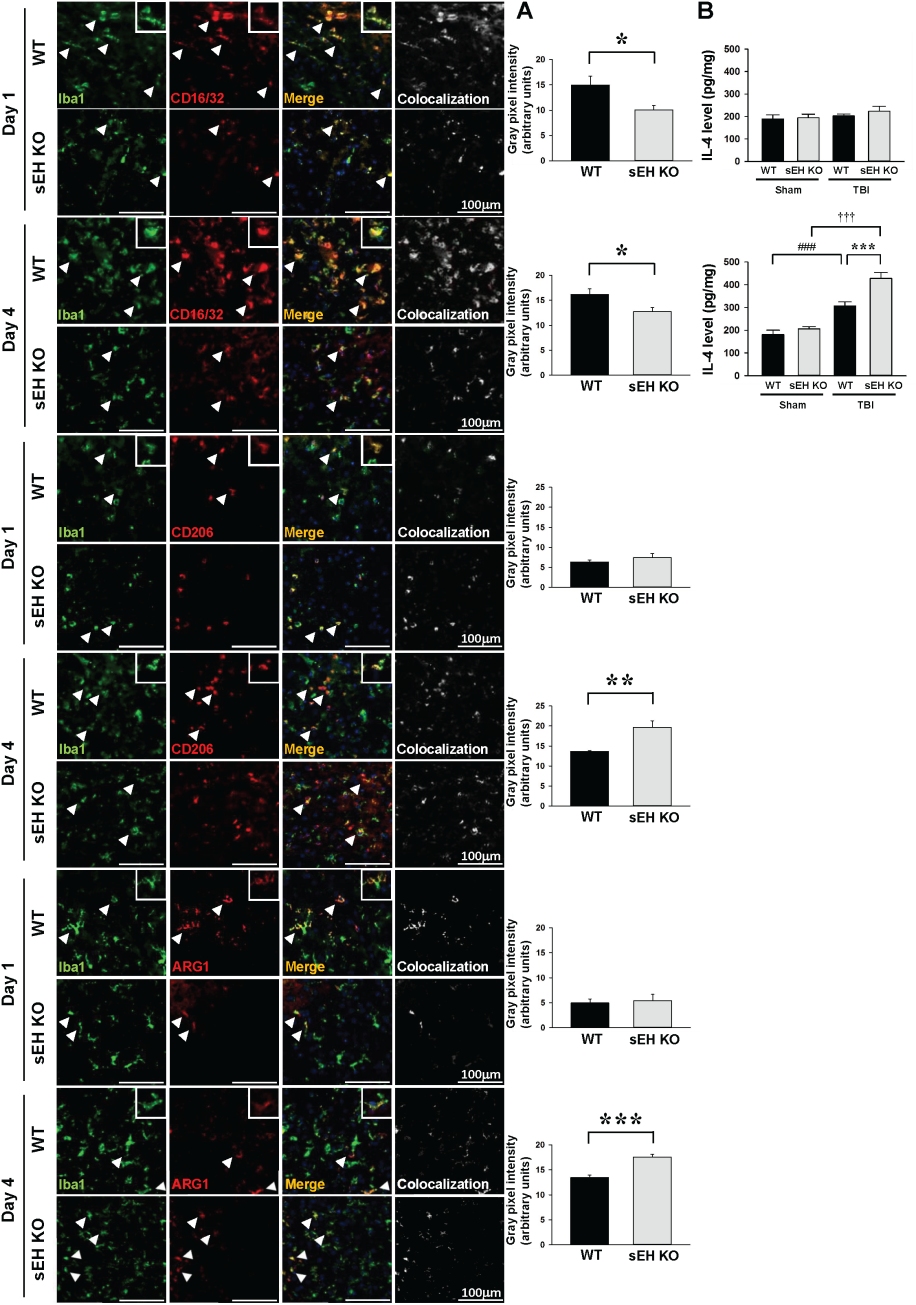
Deletion of sEH reduced proinflammatory microglia/macrophage activation and heightened anti-inflammatory microglia/macrophage response after TBI **(A)** Representative double-immunofluorescence of CD16/32 (classical activation marker; red), CD206 or arginase 1 (ARG1, alternative activation markers; red) and Iba1 (green) in the contusion margin (see the red box in the brain atlas coronal brain section in Figure 3C) of WT and sEH KO mice at 1 day and 4 days following TBI. Arrowheads indicate the cells of colocalization. The inset images represent higher magnification of the boxed region in the corresponding images. The scale bar is 100 μm. The bar graph shows the degree of Iba1 and CD16/32, CD206 or arginase 1 colocalization in gray pixel intensity. Deletion of sEH resulted in a significant decrease of CD16/32-Iba1double-positive signal at 1 day and 4 days and an increase of CD206-Iba1 or arginase-1 double-positive signal at 4 days post-TBI. **(B)** Bar graphs of IL-4 protein level as assessed by ELISA in ipsilateral hemispheres of WT and sEH KO mice at 1 day and 4 days following sham surgery or TBI. Deletion of sEH caused a significant elevation of IL-4 level at 4 day. Values are mean ± S.E.M; ^###^, ^†††^ *P* < 0.001 vs. sham group; *P < 0.05, ** *P* < 0.01, ***P < 0.001 vs. WT group (n = 5 mice / group for CD16/32, CD 206 or arginase 1 and Iba1 double stainings, Student’s *t*-test; n = 6 mice / group for IL-4 ELISA, repeated measures two-way ANOVA).

Microglial and macrophage activation involves diverse phenotypes with different physiological roles that have been historically classified as a classically activated, proinflammatory M1 phenotype or an alternatively activated, anti-inflammatory M2 phenotype [26, 27]. We next investigated whether sEH deletion affected the polarization of microglia/macrophages, using double-immunofluorescent staining with the microglia/macrophage marker Iba1 and with the marker of classically activated M1 microglia/macrophages (CD16/32) or alternatively activated M2 microglia/macrophages (CD206, arginase 1). While no CD16/32-positive microglia/ macrophages were observed in sham-operated brains (Supplementary Figure 2), CCI induced an increase of CD16/32-positive microglia/ macrophages in the contusion margin at both 1 and 4 days (Figure 6A). The CD16/32-Iba1 double-positive signal in the contusion margin was significantly decreased following sEH deletion at both 1 day (*P* = 0.035) and 4 days (*P* = 0.044; Figure 6A). For M2 markers, CD206- or arginase 1-positive microglia/ macrophages were not detectable in sham-operated brains but were detectable at both 1 day and 4 days after injury. Genetic deletion of sEH resulted in a significant increase of M2 microglia/macrophages CD206-Iba1 and arginase 1-Iba1 double-positive signals at 4 days post-injury in the contusion margin, emphasizing the anti-inflammatory role of sEH deletion (*P* = 0.007 for CD206 and *P* < 0.001 for arginase 1; Figure 6A). IL-4 induces alternative M2 activation in microglia and macrophages [28]. We therefore sought to assess whether sEH deletion influenced IL-4 expression. Consistent with the CD206 and arginase 1 immunoreactivity results, the M2-polarizing cytokine IL-4 level was significantly increased following sEH deletion at 4 days post-injury (427.6 ± 26.5 versus 307.4 ± 17.2 pg/mg protein, *P* < 0.001; Figure 6B). Thus, sEH involvement in the regulation of inflammatory response may be crucial in the development of brain damage following TBI.

### Pharmacological inhibition of sEH by AUDA ameliorates brain edema, apoptosis, and reduces neuroinflammation after TBI

**Figure 7:**
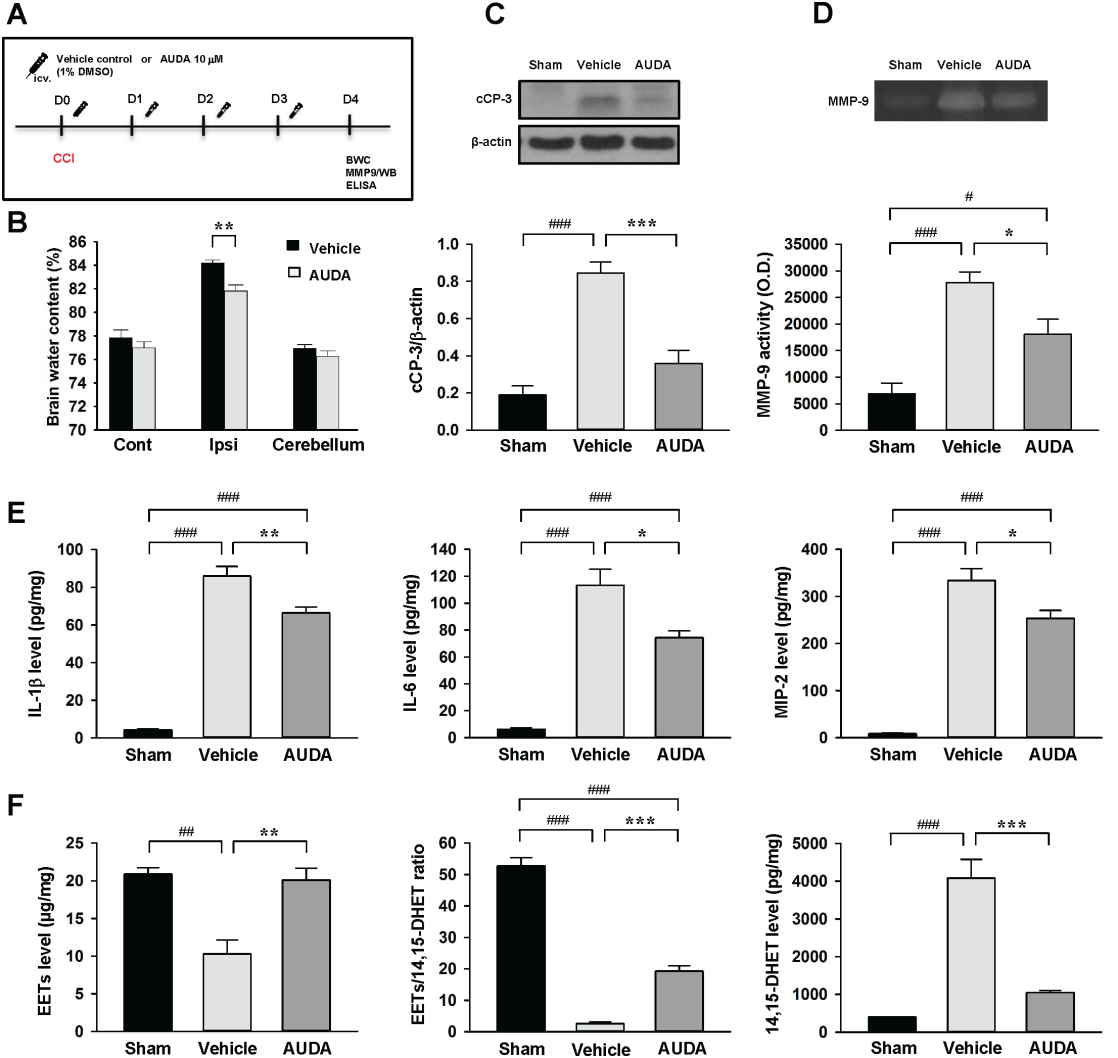
Intracerebroventricular administration of AUDA reduced brain edema, apoptosis, MMP-9 activity, and expression of inflammatory cytokines and chemokines and EET degradation after TBI **(A)** Experimental design and animal groups classification. TBI: traumatic brain injury; BWC: brain water content; MMP-9: matrix metalloproteinase-9; WB: Western blots; ELISA: enzyme-linked immunosorbent assay. **(B)** AUDA significantly decreased brain water content in the ipsilateral hemisphere compared with the WT mice. Cont: contralateral cortex; Ipsi: ipsilateral cortex. **(C)** Western blot analysis of cleaved caspase-3 and **(D)** MMP-9 activity in the ipsilateral hemisphere of sham-injured, vehicle-treated and AUDA-treated mice at 4 days following injury. AUDA treatment significantly decreased the cleaved caspase-3 level and MMP-9 activity at 4 days post-TBI. cCP-3: cleaved caspase-3. Bar graphs demonstrating **(E)** IL-1β, IL-6, MIP-2 levels and **(F)** EET level, EET/14,15 DHET ratio and 14,15 DHET protein level, as assessed by ELISA in the ipsilateral cortices of WT at 4 days following sham surgery or TBI. AUDA significantly attenuated IL-1β, IL-6, and MIP-2 protein levels compared with WT mice at 4 days post-injury. AUDA-treated mice exhibited significantly increased EETs level, elevated EET/ 14,15 DHET ratio, and reduced 14,15 DHET level compared with vehicle-treated mice at 4 days post-TBI. Values are mean ± S.E.M; ^#^*P* < 0.05, ^##^*P* < 0.01, ^###^*P* < 0.001 vs. sham group; *P < 0.05, **P < 0.01, ***P < 0.001 vs. vehicle group (n = 7 mice / group for brain water content, n = 5-7 mice / group for Western blot analysis, MMP-9 activity, cytokines and chemokines, and n = 4 mice / group for EETs and 14, 15 DHET, one-way ANOVA).

To further investigate whether pharmacological inhibition also provided protection against TBI, we treated WT mice with intracerebroventricular (i.c.v.) injection of the sEH inhibitor AUDA (10uM) for four consecutive days following CCI (Figure 7A). We chose the route of i.c.v. administration to focus on the central sEH inhibition effects. Consistent with the responses of sEH KO mice, AUDA treatment significantly reduced brain water content (81.8 ± 0.5% vs. 84.2 ± 0.3%, *P* = 0.0017; Figure 7B) and the cleaved caspase-3 level (42.4% of the vehicle-level, *P* < 0.001; Figure 7C) of the ipsilateral hemisphere compared with vehicle control treatment at 4 days post-injury. The MMP-9 activity was also significantly reduced following AUDA treatment (*P* = 0.023; Figure 7D). Similarly, the expression of IL-1β, IL-6 and MIP-2 was significantly decreased in the AUDA-treated injured brains compared with the vehicle group at 4 days (IL-1β: 66.4 ± 3.1 vs. 86.0 ± 5.1 pg/mg protein, *P* = 0.005; IL-6: 74.3 ± 5.1 vs. 113.2 ± 12.0 pg/mg protein, *P* = 0.01; MIP-2: 253.2 ± 17.0 vs. 333.9 ± 24.8 pg/mg protein, *P* = 0.02; Figure 7E). Moreover, CCI induced reductions in EET level and EET/14,15-DHET ratio, and an increase in 14,15-DHET level (all *P* < 0.01; Figures 7F). In parallel with the findings in genetic deletion of sEH, AUDA treatment resulted in significantly elevated EET level (*P* = 0.003) and decreased EET/14,15-DHET ratio (*P* < 0.001), and reduced 14,15-DHET level (*P* < 0.001; Figure 7F). These findings indicate that pharmacological inhibition of sEH using AUDA resulted in a reduction of EET degradation, accompanied by attenuation of brain edema, apoptosis and inflammatory responses following TBI.

### Pharmacological inhibition or genetic deletion of sEH attenuated LPS- or interferon-gamma-induced proinflammatory responses in cultured microglia

**Figure 8:**
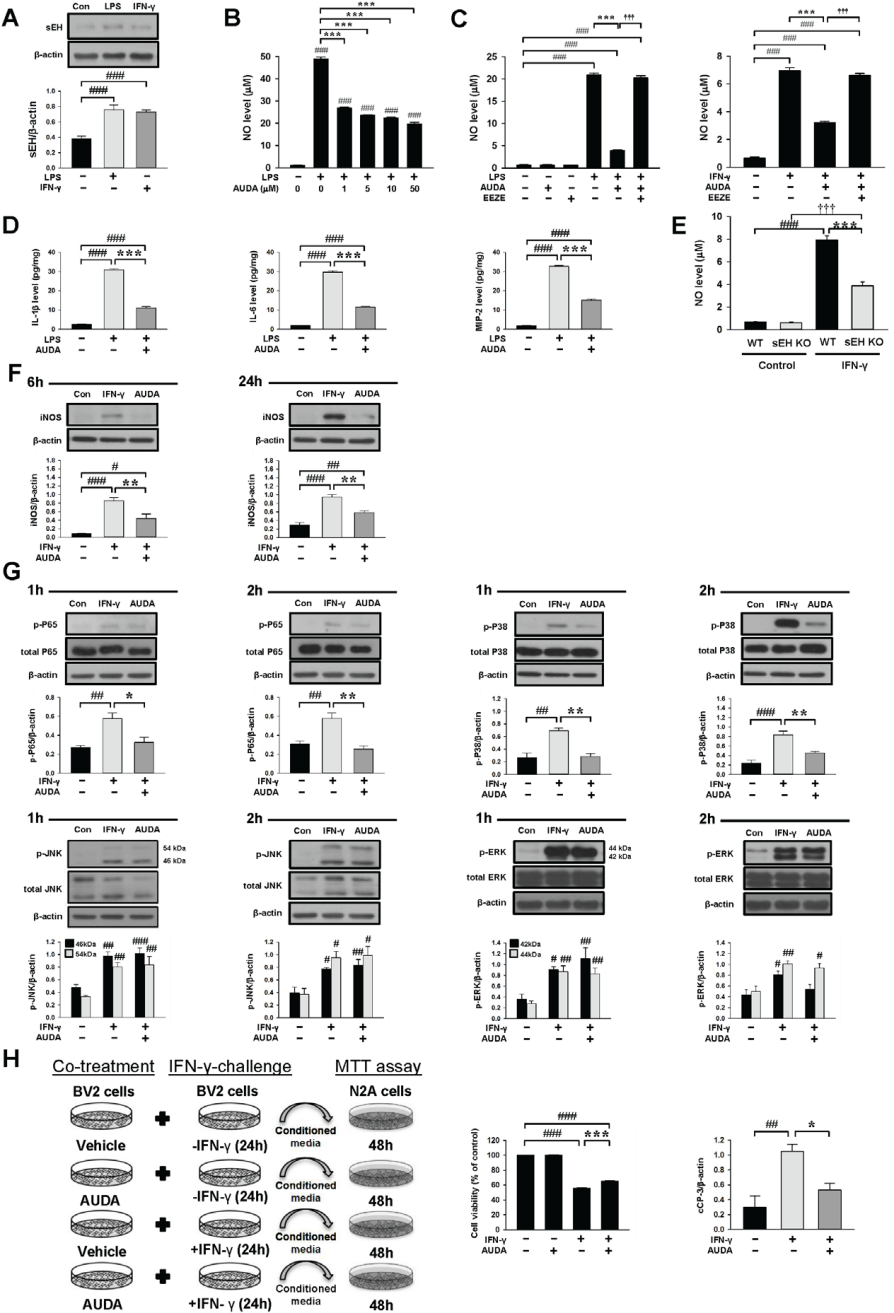
Pharmacological inhibition or genetic deletion of sEH inhibited LPS- or IFN-γ-induced inflammatory responses in cultured microglia **(A)** In BV2 microglia, both LPS and IFN-γ treatment for 24 h induced a significant increase of sEH protein level. **(B)** In BV2 microglia, co-treatment of 1, 5, 10, or 50μM AUDA with LPS for 24h significantly attenuated LPS- induced release of NO from the supernatant of microglial cultures. In primary microglia, co-treatment of 10μM AUDA with **(C)** LPS or IFN-γ for 24 h significantly reduced LPS- or IFN-γ-induced release of NO from the supernatants of microglial cultures. The protective effect of AUDA on microglial activation was completely abolished by administration of the putative pan-EET receptor antagonist 14,15-EEZE (1μM) in both LPS- and IFN-γ-stimulated primary microglia. **(D)** Co-treatment of 10μM AUDA with LPS for 24 h significantly reduced LPS-induced release of IL-1β, IL-6 and MIP-2 from the supernatants of primary microglial cultures. **(E)** In primary mouse microglial cultures, deletion of sEH significantly attenuated IFN-γ-induced release of NO from the supernatant of microglial cultures. **(F)** Representative immunoblots and bar graphs show that co-treatment of 10μM AUDA with IFN-γ significantly reduced iNOS protein levels at 6 h and 24h. **(G)** Representative immunoblots and bar graphs show that co-treatment of 10μM AUDA with IFN-γ significantly reduced IFN-γ-induced P65 and P38 phosphorylation at 1 h and 2 h, but did not affect JNK or ERK phosphorylation in primary microglia. **(H)** Experimental scheme of neuronal survival in N2A cells in response to IFN-γ-treated BV2-conditioned media with or without AUDA pretreatment. BV2 microglia were incubated with IFN-γ in the absence (IFN-γ-CM) or presence of 10μM AUDA (IFN-γ-/AUDA-CM) for 24 h. Cell-free supernatant fractions were applied to N2A cells for 48 h to evaluate the changes in cell viability and cleaved caspase-3 level. Neuronal cell death increased after exposure to IFN-γ-treated conditioned microglial media; the effect was significantly reduced by microglia pretreatment with 10μM AUDA. Western blot analysis showed that AUDA significantly reduced the cleaved caspase-3 level compared with N2A cells treated with conditioned microglia media alone. cCP-3: cleaved caspase-3. Values are presented as mean ± S.E.M of four independent experiments. ^#^*P* < 0.05, ^##^*P* < 0.01, ^###^, ^§§§^*P* < 0.001 vs. normal control; *P < 0.05, **P < 0.01, ***P < 0.001 vs. LPS/IFN-γ stimulation alone or WT group (one way ANOVA for pharmacological experiments and two-way ANOVA for gene deletion experiment).

Our *in vivo* results demonstrated that both genetic deletion and pharmacological inhibition of sEH reduced brain damage and inflammatory responses after TBI. Thus, we further used mouse BV2 microglial cells and primary cultured microglia to investigate whether sEH directly modulated microglia-mediated neuroinflammation and to elucidate the underlying molecular mechanisms. Lipopolysaccharide (LPS), a strong immunostimulant, and interferon (IFN)-γ, a cytokine that is released following TBI [29], were used to activate microglia. To establish the role of microglial sEH in microglial activation, we first determined sEH protein expression. Both LPS (100 ng/mL) and interferon-gamma (IFN-γ, 10 ng/mL) treatment for 24 h induced significant increases in sEH protein levels (2-fold) in BV2 microglial cells (both *P* < 0.001; Figure 8A). The exposure of BV2 microglia to LPS for 24 h markedly increased NO release in the culture supernatant, but co-treatment with 1, 5, 10, or 50 μM AUDA for 24 h significantly reduced LPS-induced NO production to 55%, 48%, 46% and 40% of that observed for the vehicle control group, respectively, and 10 μM AUDA provided the highest degree of anti-inflammatory action (all *P* < 0.001; Figure 8B). Therefore, the dosage of 10 μM was employed for subsequent studies. We further used primary cultured microglia to confirm the effects of AUDA on microglial activation. Incubation with both LPS (100 ng/mL) and IFN-γ (10 ng/mL) for 24 h increased NO release in the culture supernatants and co-treatment with 10 μM AUDA for 24 h significantly reduced LPS-induced NO production to 18% and IFN-γ-induced NO production to 46% of that observed for the vehicle control group(both *P* < 0.001; Figure 8C). To confirm whether the protective effect of the sEH inhibition was attributed to increased EETs, we treated LPS- or IFN-γ- stimulated primary microglia with 10 μM AUDA in the presence or absence of the putative pan-EET receptor antagonist 14,15-EEZE (1μM). 14,15-EEZE did not affect the baseline NO level but the protective effect of AUDA on microglial activation was completely abolished by administration of 14,15-EEZE in both LPS- and IFN-γ- stimulated primary microglia (Figures 8C), indicating that the salutary effect observed with sEH inhibition was specifically due to EETs. Next, we analyzed the effects of AUDA on release of the proinflammatory cytokines and chemokines, a key hallmark of microglial activation. IL-1β, IL-6 and MIP-2 levels were significantly increased in the culture media of LPS-stimulated primary microglia, and these increases were significantly decreased by treatment with 10 μM AUDA (IL-1β: 11.1 ± 0.7 vs. 30.8 ± 0.7 pg/mg, *P* < 0.001; IL-6: 11.6 ± 0.3 vs. 29.7 ± 0.7 pg/mg, *p* < 0.001; MIP-2: 15.1 ± 0.5 vs. 32.6 ± 0.5 pg/mg, *p* < 0.001, Figure 8D). We further investigated whether the lack of sEH would influence microglial activation by comparing IFN-γ-induced NO production using primary microglia derived from WT and sEH KO mice. When stimulated by 10 ng/mL IFN-γ, NO release was significantly increased in the culture supernatants of both WT and sEH KO mice by 24 h (Figure 8E). Deletion of sEH significantly reduced IFN-γ-induced NO production to 49% that observed for the WT group (*P* < 0.001; Figure 8E), suggesting that sEH deletion suppressed IFN-γ-stimulated microglial activation.

As AUDA was found to inhibit NO production, we examined the effect of AUDA on inducible nitric oxide synthase (iNOS) protein expression. The iNOS protein levels were markedly up-regulated after 6 and 24 h of LPS or IFN-γ treatment, and AUDA significantly attenuated iNOS protein expression in LPS-stimulated primary microglia at 6 h (69% of vehicle-level, *P* = 0.002) and 24 h (50% of vehicle-level, *P* = 0.008) and in IFN-γ-stimulated primary microglia at 6 h (52% of vehicle-level, *P* = 0.007) and 24 h (62% of vehicle-level, *P* = 0.001), respectively (Supplementary Figure 3A & Figure 8F). Collectively, these results support the notion that sEH functions as an important regulator of microglial activation.

**Figure 9:**
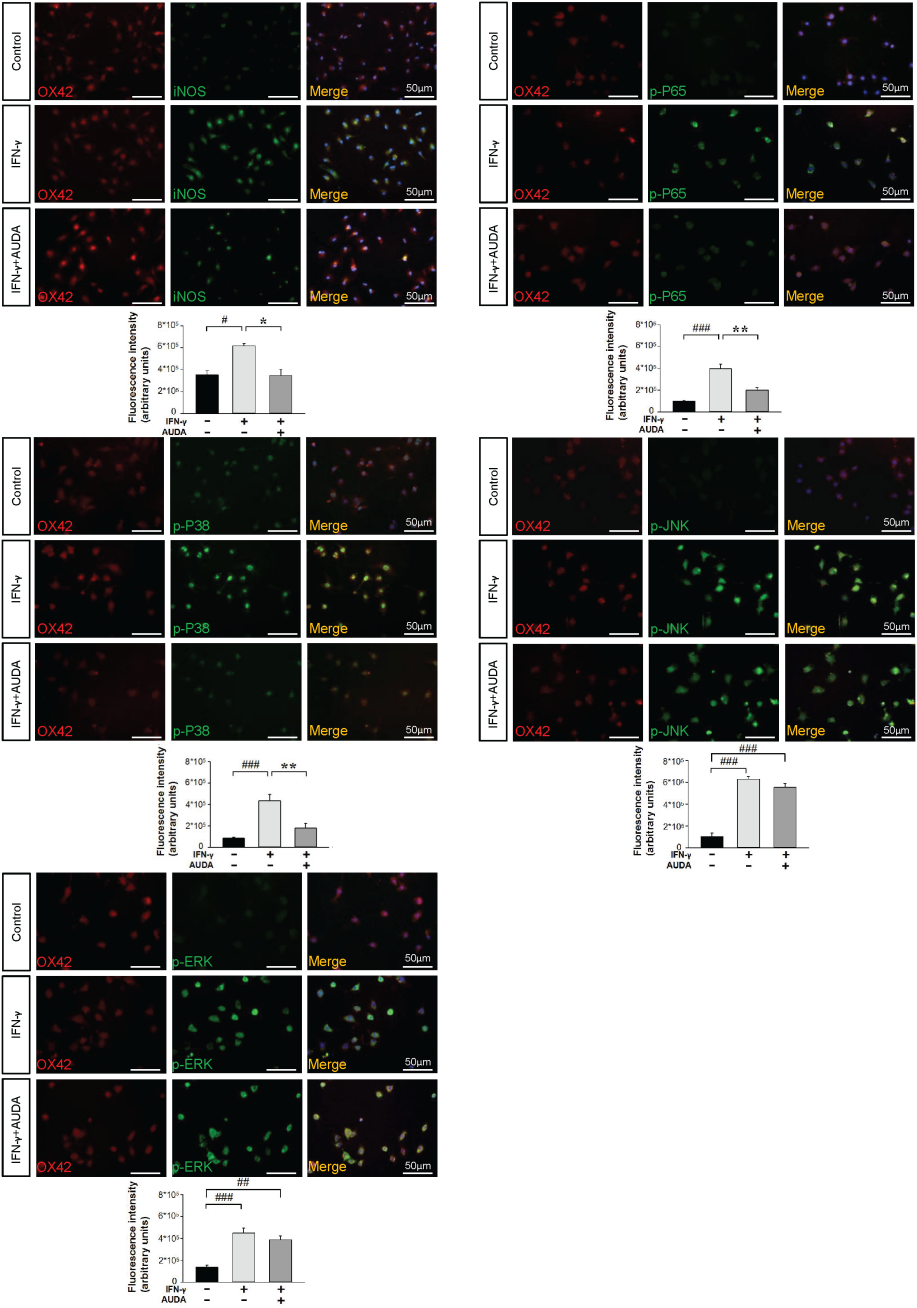
AUDA inhibited IFN-γ-induced iNOS expression and phosphorylation of P65 and P38 in primary microglia cultures Immunolabeling of iNOS (green), phospho-P65 (green), phospho-P38 (green), phospho-JNK (green), phospho-ERK p44/42 (green) with OX42 (red), a marker of microglia. Co-localization is shown by yellow labeling. Cells were stained with DAPI (blue) to show all nuclei. Bar graphs show thatco-treatment of 10μM AUDA with IFN-γ significantly reduced immunoreactivity for iNOS at 24 h, phospho-P65, phospho-P38 at 2 h but had no effect on phospho-JNK or phospho-ERK p44/42 immunoreactivity at 2 h. Values are presented as mean ± S.E.M of four independent experiments. ^#^*P* < 0.05, ^##^*P* < 0.01, ^###^*P* < 0.001 vs. normal control; *P < 0.05, **P < 0.01 vs. IFN-γ stimulation alone (one-way ANOVA). The scale bar is 50 μm.

We next investigated whether AUDA influenced the activation of mitogen-activated protein kinases (MAPKs)-NF-κB, a major signaling pathway that induces a variety of proinflammation mediators in microglia during acute brain injury [30]. Activation of NF-κB, as indicated by phosphorylation of P65 at serine 536, was observed at 1 and 2 h following LPS or IFN-γ stimulation (all *P* < 0.01; Supplementary Figure 3B & Figure 8G). Co-treatment with 10 μM AUDA significantly attenuated the LPS-induced increased levels of p-P65 Ser536 at 1 h (69% of vehicle-level, *P* = 0.002) and 2 h (73% of vehicle-level, *P* < 0.001), and the IFN-γ-induced increased levels of p-P65 Ser536 at 1 h (69% of vehicle-level, *P* = 0.014) and 2 h (73% of vehicle-level, *P* = 0.001) (Supplementary Figure 3B & Figure 8G). Next, we evaluated the inhibitory effects of AUDA on LPS- or IFN-γ-induced activation of MAPKs, including P38, jun amino-terminal kinases (JNK) and extracellular signal-regulated kinases p44/42 (ERK). Stimulation of microglia with both LPS and IFN-γ for 1 and 2 h resulted in rapid activation of P38, JNK and ERK. Co-treatment with 10 μM AUDA significantly reduced LPS-induced P38 phosphorylation at 1 h (69% of vehicle-level, *P* < 0.001) and 2 h (73% of vehicle-level, *P* = 0.011), and IFN-γ-induced P38 phosphorylation at 1 h (69% of vehicle-level, *P* = 0.002) and 2 h (73% of vehicle-level, *P* = 0.005) (Supplementary Figure 3B & Figure 8G). However, ERK and JNK phosphorylation was not affected (all *P* > 0.05; Supplementary Figure 3B & Figure 8G). Similarly, AUDA significantly attenuated immunoreactivity for iNOS (*P* = 0.018) at 24 h, phospho-P65 (*P* = 0.002), phospho-P38 (*P* = 0.007) at 2 h but had no effect on phospho-JNK or phospho-ERK p44/42 at 2 h following IFN-γ stimulation in primary microglia (Figure 9). Together, these results suggest that AUDA suppress the P38 signaling pathway and NF-κB activation, resulting in the inhibition of microglial activation and proinflammatory responses.

Activated microglia are thought to contribute to delayed neuronal death after TBI by releasing neurotoxic mediators [6]. Accordingly, we further investigated whether inhibition of sEH in microglia conferred protection against microglial-induced neuronal injury by measuring the viability of N2A cells exposed to LPS-treated or IFN-γ-treated conditioned media from BV2 microglia for 2 days in the presence or absence of AUDA (Supplementary Figure 3C & Figure 8H). Conditioned media from LPS-stimulated microglia (LPS-CM) or IFN-γ-stimulated microglia (IFN-γ-CM) significantly decreased N2A cell viability to 53.4 ± 0.5% (*p* < 0.001) or 55.5 ± 0.5% (*p* < 0.001) of the control-level, respectively. However, the viability of N2A cells stimulated with LPS-treated (LPS/AUDA-CM) or IFN-γ–treated (IFN-γ/AUDA-CM) conditioned microglia media from the 10μM AUDA treatment group markedly increased to 62.4 ± 0.8% (*p* < 0.001) and 65.2 ± 0.2% (*p* < 0.001), respectively (Supplementary Figure 3C & Figure 8H). The cleaved caspase-3 levels in N2A cells treated with LPS/AUDA-CM or IFN-γ-/AUDA-CM were also significantly decreased to 40.2% (*P* = 0.035) or 50.7% (*P* = 0.037) of the vehicle-level, respectively (Supplementary Figure 3C & Figure 8H). Taken together, these results indicate that the protective effect of AUDA on neuronal viability is at least partly conferred through the inhibition of microglial-induced injury.

## DISCUSSION

In this study, we show that genetic deletion of sEH improved long-term behavioral outcomes and attenuated brain edema in mice subjected to TBI. Brain tissue damage, apoptosis, and BBB disruption were also reduced in sEH KO mice. Mechanistically, sEH deletion reduced microglial/macrophage activation, neutrophil infiltration, protein expression of proinflammatory mediators and MMP-9 activity. Similar to sEH gene deletion, pharmacological inhibition of sEH using AUDA reduced brain edema, apoptosis and inflammatory responses in mouse TBI. In the *in vitro* studies, AUDA attenuated LPS-stimulated or IFN-γ-stimulated NO production in both primary microglia and the BV2 cell line, which was associated with reduced activation of P38 and NF-κB signaling in both LPS-stimulated and IFN-γ-stimulated primary microglia. Deletion of sEH also reduced IFN-γ-stimulated NO production in mouse primary microglia. Conditioned media from LPS-stimulated or IFN-γ-stimulatedBV2 cells caused death in N2A cells, but treating LPS-stimulated or IFN-γ-stimulatedBV2 cells with AUDA reduced neuronal cell death induced by microglial conditioned media. The current study extends upon previous findings of the protective effects of sEH inhibition on behavioral recovery following TBI in mice and provides new evidence of the role of microglia-induced inflammation in the neuroprotective effects of sEH inhibition in TBI.

We showed that TBI induced upregulation of sEH protein and that sEH was expressed in microglia in the injured brain. This was associated with a decrease in EET level and an increase in 14,15 DHET level. Our data also demonstrated upregulation of sEH protein expression levels in LPS- and IFN-γ-stimulated microglia, suggesting that inhibition of sEH would be beneficial because it likely increases and prolongs the functional effects of anti-inflammatory EETs in this proinflammatory condition following TBI. The sEH gene promoter region contains recognition sites for a number of transcription factors including NF-κB and activator protein [31], both of which respond to inflammation and are activated following TBI [32–34]. Indeed, our data are in line with previous studies showing an elevated cerebral sEH protein level in rodent models of brain damage including cerebral ischemia [12], Parkinsonism [16], and seizures [17]. We showed that TBI induced increased sEH expression at 1 h. As previous studies have demonstrated that rodent TBI induced fast activation of NF-κB as early as 15 min [32] and increased c-fos and c-Jun mRNA expression (components of the AP-1 complex) at 5 min after injury [33, 34], it is possible that TBI-induced activation of NF-κB and AP-1 contributes to the early elevation in sEH expression.

Accumulating evidence has documented the effect of sEH inhibition in protection against various models of brain damage [16, 17, 19, 20]. Both gene deletion and pharmacological inhibition of sEH also reduce functional and histological damage following experimental cerebral ischemia [19, 20]. Although gene deletion of sEH reduced motor and working memory deficits caused by mouse TBI [21], it remains undetermined whether it attenuated the extent of neuronal damage, and the precise mechanisms of the protective effect have not been investigated. Thus, the current data confirm and extend upon previous reports showing a beneficial effect of sEH inactivation in improving long-term functional and histological outcomes following TBI. Specifically, we expand upon previous findings by demonstrating that sEH inhibition reduced microglial activation and contributed to improved neuronal survival. We showed that the level of EETs and the ratio of EETs to 14,15-DHET in the injured brain were significantly increased in sEH KO mice and AUDA-treated mice and that administration of the 14,15-EET antagonist 14,15-EEZE abolished the anti-inflammatory effect of AUDA in cultured microglia, suggesting that the protection mediated by sEH inhibition is mainly due to augmenting endogenous 14,15-EET. Taken together, these data demonstrate that selectively increasing cerebral EET concentrations by sEH inhibition not only improved neurobehavioral outcomes but also reduced brain tissue damage following TBI in mice, thereby providing evidence in support of sEH inhibitors as a potential therapeutic intervention for neuroprotection after TBI.

Brain edema is one of the major prognostic factors following TBI and may cause increased intracranial pressure, decreased cerebral perfusion pressure and eventually reduced cerebral blood flow with impaired glucose and oxygen delivery to the brain tissue [5]. In the present study, we showed that both gene deletion and pharmacological inhibition of sEH attenuated brain edema, MMP-9 activity and the expression of cytokines and chemokines. Deletion of sEH also reduced BBB disruption, neutrophil infiltration, and microglial activation. Following TBI, microglial activation impairs BBB function and causes brain edema due to the release of various molecules including oxygen reactive species, proteolytic enzymes, and inflammatory cytokines [6, 24]. The increased levels of MMPs, particularly MMP-9, disrupt the basal lamina proteins and degrade the tight junction complexes, also resulting in BBB breakdown [24]. In addition, excessive recruitment of neutrophils in the brain causes the release of inflammatory mediators and MMPs, which may further activate microglia, thus impairing BBB integrity and exacerbating brain edema formation [35]. Our results are in agreement with published data showing that deletion of the sEH gene reduced hydrocephalus, vascular inflammation and brain edema after experimental subarachnoid hemorrhage [36]. To clarify whether the effect of sEH inhibition on TBI-induced brain edema is reduced cerebral inflammation, we injected the sEH inhibitor, AUDA, directly into the lateral ventricle. The parallel results obtained with AUDA following i.c.v. administration were consistent with the results of the experiments in sEH KO mice. Therefore, our results suggest that the protective effect on brain edema is mainly attributed to a reduction in cerebral inflammatory responses in the injured brain. Nevertheless, it remains undetermined whether sEH may affect BBB integrity via activating peripheral immune cells. Indeed, recent studies have reported that 8,9-EET inhibited LPS-induced B cell activation both *in vivo* and *in vitro* [37]. Additionally, pharmacological inhibition of sEH significantly reduced the number of neutrophils, alveolar macrophages, and lymphocytes in bronchoalveolar lavage fluid in tobacco smoke-exposed rats [38]. TBI induces a systemic inflammatory response including increased circulating neutrophil counts and plasma levels of inflammatory mediators [39], both of which contribute to TBI-induced brain edema [35]. Therefore, it is important to explore the peripheral effect by which sEH inhibition protects against TBI in future studies.

Following TBI, BBB disruption may occur via disruption of tight junction proteins (paracellular transport), increases in transcellular transport, mainly due to dysregulation of endothelial receptors and intracellular transporters, and loss of endothelial cells [40]. We demonstrated that deletion of sEH reduced BBB permeability but did not affect expression of tight junction proteins, suggesting that the protection of BBB integrity in sEH-KO mice may be via mechanisms other than increasing tight junction protein expression such as reducing transcellular transport or promoting endothelial cell survival. This explanation is supported by a previous study showing that experimental TBI induced an early increase in BBB permeability despite the integrity of the endothelial tight junctions [41]. Also, ultrastructural studies have shown that caveolae at endothelial cells are increased in vessel segments showing BBB disruption following rodent brain injury [42]. Furthermore, previous studies have demonstrated that post-translational modification of tight junction proteins without changing protein expression may alter junctional protein function and thus affect BBB integrity [43]. Our current results could not exclude the possibility that deletion of sEH may maintain BBB integrity via mechanisms such as reducing transcellular transport, promoting endothelial cell survival or attenuating post-translational modification of tight junction proteins. Therefore, the mechanisms by which sEH affects the BBB integrity remains to be elucidated in future studies.

Although recent studies have demonstrated that gene deletion or pharmacological inhibition of sEH elicits anti-inflammatory effects *in vivo* [12, 17, 44], the direct contribution of sEH inhibition on the regulation of NF-κB-dependent microglial activation has remained unclear. Microglial activation plays a key role in secondary injury after TBI by releasing reactive oxygen species as well as other neurotoxic molecules [6]. Our *in vivo* results demonstrated that deletion of sEH significantly reduced the number of activated microglia (hypertrophic and bushy morphologies) in the injured cortex at both 1 and 4 days after TBI. Both gene deletion and pharmacological inhibition attenuated the expression of inflammatory mediators (e.g., IL-1β, IL-6, MIP-2, and MCP-1) in the injured brain at these two time-points. These findings are in line with previous studies in which deletion or pharmacological inhibition of sEH reduced microglial activation and cytokine expression in animal models of cerebral ischemia [12], spinal cord injury [44], and seizure [17]. Notably, we further used two *in vitro* models of microglial activation (LPS and IFN-γ) in the BV2 microglia cell line and in primary microglia to directly evaluate the effects of sEH inhibition or deletion on microglia. We found that AUDA treatment reduced iNOS expression, the release of NO, IL-1β, IL-6 and MIP-2, and the activation of NF-κB and P38 in primary microglial cultures in response to LPS or IFN-γ stimulation. Also, deletion of sEH attenuated the release of NO in IFN-γ–stimulated primary microglial cultures. Our data advance previous observations supporting the hypothesis that sEH inhibition directly suppressed microglial activation by attenuating the P38-NF-κB pathway. However, we did not distinguish microglia and macrophages in the *in vivo* experiment as there is no known cell-surface marker to distinguish brain-resident microglia from blood-derived macrophages. Therefore, we cannot exclude the involvement of blood-derived macrophages regarding the anti-inflammatory effect of sEH inhibition following mouse TBI as it is difficult to make the separation of brain resident microglia and blood-derived macrophages by immunohistology employed in this study. Methods to distinguish microglia from macrophages mainly depend on relative marker expression by flow cytometry based on levels of CD45 expression or generating bone marrow chimeras [45]. Another limitation of this study is that we used primary microglial cultures from rats instead of from mice as the number of microglia cells obtained from postnatal mouse brains was not large enough for Western blot analysis. However, in line with the fact that deletion of sEH in our study reduced microglial/macrophage activation and neuronal death following mouse TBI and that AUDA attenuated the neuronal cell death induced by microglia-conditioned media in the *in vitro* experiment, our observations suggest that the protection of damaged neurons by sEH inhibition was mediated by inhibition of microglia/macrophage-derived proinflammatory factors in the injured brain.

Previous studies have shown that MAPKs play critical roles in the cerebral inflammatory responses during acute brain injury [30, 46]. MAPK signaling consists of P38 MAPK, JNK and ERK and is involved in microglial activation. Activation of MAPK signaling contributes to activation of transcriptional factors, including NF-κB, resulting in the upregulation of many proinflammatory genes (e.g., cytokines, chemokines, and iNOS) [30, 46]. These proinflammatory mediators can then further activate MAPKs and NF-κB, forming a positive feedback loop to amplify inflammatory signals. In the present study, AUDA attenuated phosphorylation of P38 but not JNK or ERK induced by LPS or IFN-γ, indicating that the P38-MAPK pathway could represent a molecular target for sEH inhibition and thus mediate its anti-inflammatory properties in activated microglia. Our results are in accordance with a previous study demonstrating that EETs exerted anti-inflammatory effect by reducing the phosphorylation level of P38-MAPK in the culture of human bronchi [14]. However, pharmacological sEH inhibition has also been reported to inhibit the phosphorylation (activation) of JNK in human monocytes stimulated by LPS [47]. As activation of the MAPK pathways depends on the cell type, stimuli, duration of the stimulus as well as the cellular conditions [48], the inability of AUDA to affect the JNK pathway in microglia in our study may be due to different cell types. Another possibility for the selective inhibition of P38 MAPK is that, compared to P38, JNK signaling is more potent in regulating microglial survival [49], which might make JNK signaling more difficult to suppress. In addition, some studies show that EETs provide anti-inflammatory effects by activating PPARγ signaling [14, 50]. STAT1/3 signalings are other important pathways involved in microglia-induced inflammation. STAT1/3 pathways have been shown to be involved in the inflammatory signaling cascades triggered by inflammatory stimuli such as LPS, IFN-γ and other cytokines [51]. Activation of STAT 1 and 3 were also observed following rodent TBI [52]. Moreover, previous research indicated that exogenous EET administration inhibited STAT 3 activation *in vitro* [53]. Thus, the involvement of PPARγ and STAT1/3 signalings in the anti-inflammatory effects of sEH inhibition needs further investigation. Also, clarification of the role of MAPKs signaling *in vivo* is also one of our future directions.

Important considerations regarding the interpretation of our data are the multiple beneficial effects of EETs and sEH inhibition in the brain in addition to anti-inflammatory effects. Therefore, the improvement in functional or histological outcomes in the current study may result from other beneficial effects of sEH inhibition. For example, EETs and sEHIs have been shown to suppress the reactive oxygen species (ROS) and apoptosis [11, 54, 55], both of which are known to exacerbate TBI. Furthermore, EETs and sEH inhibitors are linked to angiogenesis and regulation of cerebral blood flow [11, 54, 55]. Also, a recent study demonstrated that EETs stimulated astrocyte-derived brain derived neurotrophic factor [13], an important neurotrophic factor for neuroprotection. Whether the enhancement of any of these actions by sEH inhibition contributes to neuroprotection against TBI needs to be determined. Additionally, we chose the pretreatment regimen and i.c.v. administration to allow maximal efficacy of central sEH inhibition. To explore the clinical relevance of sEH inhibition against TBI, the study of post-treatment with different doses and times of sEH inhibitors will be investigated by our group.

Both astrocytes and microglia are activated following TBI and are involved in TBI-induced neuroinflammatory processes [56]. However, contrary to the detrimental effect in secreting inflammatory factors, astrocytes can elicit several protective actions, which can promote repair or reduce damage after TBI. For example, astrocytes produce anti-inflammatory factors to inhibit microglial activation in injury states [57]. Also, astrocytes secrete neurotrophic factors, including as brain-derived neurotrophic factor and vascular endothelial factor, both of which can protect neurons from ischemic injury [13, 58]. Therefore, this study focused on the effect of sEH deletion/inhibition on microglial activation. Nevertheless, it is also possible that astrocytes are involved in the anti-inflammatory action of sEH deletion/inhibition.

sEH has two enzyme activities: the C-terminal hydrolase and N-terminal phosphatase activity [59]. The current study aimed to investigate the effect and molecular mechanisms of sEH hydrolase in the regulation of TBI-induced neuroinflammation. Thus, we used AUDA to inhibit the sEH hydrolase activity without affecting the phosphatase activity [60]. However, the phosphatase activity of sEH may also be involved in the pathogenesis of TBI. The phosphatase domain of sEH has been shown to negatively regulate VEGF-mediated endothelial NOS (eNOS) activity *in vivo* [61] and Akt-AMPK-mediated eNOS *in vitro* [62]. Since eNOS activity is implicated in mechanisms of neuronal injury and cerebral blood flow changes following brain injury [63], the sEH phosphatase activity may also participate in the pathogenesis of TBI.

**Figure 10:**
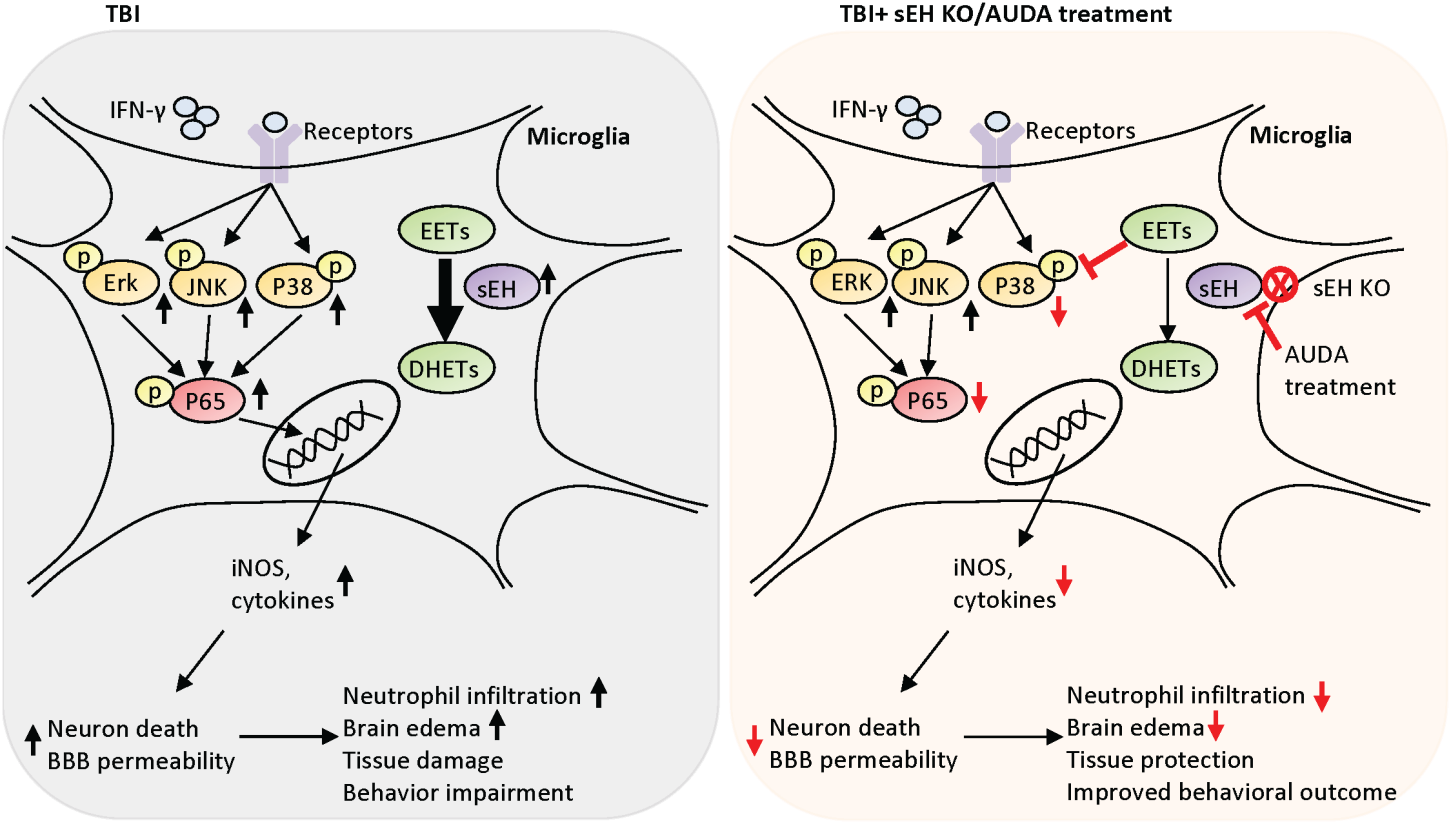
Schematic diagram of the mechanisms involved in sEH deletion or inhibition-induced protection in TBI mice Deletion of sEH gene improved behavioral outcomes, and attenuated brain edema, neuronal death, brain tissue damage and BBB disruption. sEH deletion also reduced microglial activation, neutrophil infiltration, protein expression of proinflammatory mediators. In parallel, pharmacological inhibition of sEH using AUDA reduced brain edema, apoptosis and inflammatory responses in mouse TBI. In the microglial cells, AUDA attenuated LPS-stimulated or IFN-γ-stimulated NO production, and reduced activation of P38 and NF-κB signaling. Treating LPS- or IFN-γ-stimulated microglia cells with AUDA reduced neuronal cell death induced by microglial conditioned media. Inhibition of sEH may protects against TBI by modulating the cytotoxic functions of microglia.

In summary, our study shows that gene deletion or pharmacological inhibition of sEH protects against TBI (Figure 10). This neuroprotective effect is at least in part mediated by inhibition of P38 and NF-κB signaling in activated microglia, thereby suppressing the upregulation of inflammatory mediators. We conclude that sEH inhibition after TBI can reduce microglial activation to suppress inflammation and reduce subsequent neuronal death. Our findings suggest that sEH can be a potential therapeutic target to reduce brain damage and to improve functional outcome in survivors of TBI.

## MATERIALS AND METHODS

### Animals

All the experimental protocols were approved by the Institutional Animal care and Use Committee at Cheng Hsin General Hospital (Animal permit number CHGH-102-18), and all animals were cared for in accordance with the Guide for the Care and Use of Laboratory Animals published by the US National Institutes of Health (NIH Publication No. 85-23, revised 1996). Six- to eight-week-old male sEH KO mice were purchased from Jackson Laboratory (Bar Harbor, Maine, USA). Six- to eight-week-old male C57BL/6 wild type (WT) mice were ordered from BioLASCO (Taipei, Taiwan) as controls. Mice were allowed free access to water and maintained on a 12-h/12-h dark cycle at a controlled temperature (22-25°C) and humidity (40-60%).

### Cell culture

#### Cell line cultures

The neuroblastoma neuro-2A (N2A) cell line and the mouse microglial BV2 cell line were cultured as previous described [64, 65]. Briefly, N2A and microglial BV2 cell lines were cultured in Dulbecco’s modified Eagle’s media (DMEM; Gibco, Bethesda, MD, USA) supplemented with 10% heat-inactivated fetal bovine serum (FBS; Gibco), 100 U/mL penicillin and 100 μg/mL streptomycin in a humidified atmosphere of 5% CO_2_ at 37°C.

#### Primary microglia culture

Rat primary microglia cultures were prepared from the cerebral cortex of postnatal day 7 (P7) Wistar rats similar to a previous report [66]. Primary mouse microglia culture was prepared from the cortices of P7 WT and sEH KO mice. Briefly, cortices were chopped and digested in 20 U/ml papain for 40 min at 37°C. The cells were plated with DMEM supplemented with 10% heat-inactivated FBS, 100 U/mL penicillin and 100 μg/mL streptomycin. After 2 weeks, the microglial cells were separated from the astrocytes by shaking at 200 rpm for 1 h (37°C). Non-adhered cells were eliminated, and microglial cells were re-plated on poly-L-lysine (Sigma)-coated wells (1×10^5^ cells per well) in DMEM and 5% inactivated horse serum and used for the experiments 24 h later. The purity of cultured microglia was higher than 99% under these conditions, as verified by GFAP and Iba1 staining.

### Culture drug treatment

BV2 microglia or primary rat/mouse microglia were stimulated with either 100 ng/mL LPS or 10 ng/mL IFN-γ in the absence or presence of varying concentrations of AUDA (Cayman, Ann Arbor, MI, USA) or 14,15- epoxyeicosa-5(Z)-enoic acid (EEZE; 1 μM, Cayman) for 1 h, 2 h, 6 h or 24 h. To collect the conditioned media, BV2 microglia were plated and incubated with LPS or 10 ng/mL IFN-γ in the absence (LPS–CM; IFN-γ–CM) or presence of 10 μM AUDA (LPS/AUDA–CM; IFN-γ/AUDA–CM) for 24 h. Cell-free supernatant fractions were applied to N2A cells for 48 h to evaluate the changes in cell viability and related parameters. Neuronal cell death was assessed by 3-[4,5-dimethyl-2-thiazolyl]-2,5-diphenyl-2-tetrazolium bromide (MTT) assays. The experiments were repeated four or five times with different batches of cultures.

### Experimental protocol

Mice were randomized into different treatment groups by using computer-generated random numbers. All outcome measurements and analyses described below were performed in a blinded manner. Three studies were conducted. The first study examined the temporal profile and cellular localization of sEH expression after TBI. Assessment included Western blots (n = 5-6/group) and double immunofluorescence labeling (n = 6/group). The second study evaluated the neuroprotective and anti-inflammatory effects of sEH gene deletion. The assessments were as follows: 1) behavioral tests (n = 12/group); 2) histology (n = 6-7/group); 3) brain water content and Evans blue dye extravasation (n = 6-7/group) and 4) Western blot analysis, MMP-9 zymography (n = 6-7/group) and ELISA (n = 4-7/group). The third evaluated the neuroprotective and anti-inflammatory effects of AUDA, a selective sEH inhibitor, which has been widely used in experimental studies [19]. AUDA (10 μM in 0.5μL of 1% DMSO) or equal volume of vehicle (1% DMSO) was i.c.v. injected 30 min before CCI and subsequently daily for 3 days (-30 min, 24 h, 48 h and 72h). The assessments were as follows: 1) brain water content (n = 7/group). 2) Western blot analysis, MMP-9 zymography (n = 5-7/group) and ELISA (n = 4-7/group). The 4-dose regimen was chosen because inflammatory-related signals peak between 1 and 4 days after CCI and decline thereafter [67].

### Controlled cortical impact injury

Adult mice (8-12 weeks, 22-28 g) were subjected to CCI injury as previously described [68]. Briefly, mice were anesthetized with sodium pentobarbital via intraperitoneal injection (65 mg/kg; Rhone Merieux, Harlow, UK) and a 5-mm craniotomy over the right parietal cortex was made using a dental trephine drill, centered on the bregma, and 0.1 mm lateral to the midline. Injury was produced using a pneumatic piston with a 2.5-mm rounded metal tip (4 m/sec velocity, 2-mm deformation depth). The body temperature of the mice was maintained at 37.0 ± 0.5°C using a heated pad throughout the surgery and recovery period. Sham-operated mice underwent the same procedure as injured mice, except for CCI.

### Intracerebroventricular injection

AUDA (Cayman, 10 μM in 0.5μL of 1% DMSO) or equal volume of vehicle (1% DMSO) was intracerebroventricularly injected at 30 min before CCI as previously described [69]. Briefly, a 30-gauge needle attached to a Hamilton syringe was inserted into the lateral ventricle (0.5 mm posterior to the bregma, 1 mm right lateral to the midline, and 2 mm in depth). Then, AUDA or vehicle was infused with an infusion pump for 10 min at a rate of 0.05 μL/min. The needle was removed from the infusion after 20 min to prevent reflux, and the CCI surgery was performed immediately thereafter. AUDA was then administered daily for another 3 days (30 min before CCI, day 1, day 2 and day 3) to continuously maintain the sEH inhibition effect after CCI.

### Behavioral testing

Behavioral testing was performed before and at 1, 4, 7, 14, 21, and 28 days after CCI. Mice were pre-trained for both Rotarod and beam walking tests for 3 days.

#### Modified neurological severity score

The mNSS provides an index of motor, sensory, reflex, and balance ability [70]. A score of 0 corresponded to the inability to perform any test and absence of the tested reflex; thus, the higher the score, the more severe the injury. Neurological function was graded on a scale of 0-18 (normal score, 0; maximal deficit score, 18).

#### Rotarod test

An accelerating Rotarod was used to measure motor function and balance [71]. Briefly, the Rotarod speed was slowly increased from 6 rpm to 42 rpm within 7 min, and the time when the mouse fell from the Rotarod was recorded.

### Beam walking test

The test was used to assess motor function and coordination by measuring the ability of mice to cross an elevated beam [71]. The time for the mouse to traverse the beam (not to exceed 60 s) and its hindlimb performance as it crossed the beam (based on a 1 to 7 rating scale) were recorded. A score of 7 was given when animals traversed the beam with two or less footslips; 6 was given when animals traversed the beam with less than 50% footslips; 5 was given for more than 50% but less than 100% footslips; 4 was given for 100% footslips; 3 was given for traversing the beam with the affected limb extended and not touching the surface of the beam; 2 was given when animals were able to balance on the beam but not traverse it; 1 was given when animals could not balance on the beam. For the Rotarod and beam walking tests, three measurements per trial were recorded 1 h before CCI (baseline) and at 1, 4, 7, 14, 21, and 28 days post-CCI.

### Body weight measurement

Body weight was measured 1 h before CCI (baseline) and at 1, 4, 7, 14, 21, and 28 days post-CCI using a digital scale.

### Brain water content

Brain edema was assessed by measuring brain water content as a result of BBB breakdown after CCI. After decapitation (under anesthesia) at day 1 and day 4 post-injury, the ipsilateral and contralateral cortex (in a 4-mm coronal section, 2 mm from the frontal pole) and the cerebellum (as internal control) were weighed (wet weight), dried at 100°C for 24 h, and reweighed (dry weight). Water content was determined as [(wet weight-dry weight)/wet weight] ×100% [72].

### Blood-brain barrier permeability

A 2% solution of Evans blue in normal saline (4 mL/kg of body weight) was injected into the tail vein and circulated for 1 h at day 1 and day 4 post-injury. The mice were then transcardially perfused with phosphate-buffered saline (PBS), and the ipsilateral hemisphere samples were homogenized in 1 mL of 60% trichloroacetic acid via sonication. After centrifugation at 4500 rpm for 15 min at 4°C, the supernatants were diluted with ethanol (1:4). The absorbance of each supernatant for the Evans blue dye was measured at 620 nm using a spectrophotometer (Genequant 1300, GE Healthcare, UK). EB concentrations were calculated and expressed as μg/g brain tissue against a standard curve.

### Tissue processing and histology

Mice were transcardially perfused by 0.9% sodium chloride following terminal anesthesia with sodium pentobarbital (80 mg·kg^-1^, ip) at day 1 and day 4 for cresyl violet histology, FJB staining, TUNEL, and immunostaining, or at day 28 for cresyl violet staining. Brains were removed, post-fixed in 4% paraformaldehyde overnight, cryoprotected with 30% sucrose, and then sectioned coronally at 10 μm from the level of the olfactory bulbs to the visual cortex.

### FJB staining

FJB (Chemicon, Temecula, CA, USA) is a polyanionic fluorescein derivative that binds with high sensitivity and specificity to degenerating neurons. Briefly, sections were rehydrated in graded ethanol solutions (100 and 70%, 5 min each) and distilled water, incubated in 0.06% KMnO_4_ for 30 min, rinsed in distilled water for 2 min, incubated in a 0.001% solution of FJB for 30 min, and observed under a fluorescence microscope (Olympus BX-51; Olympus, Tokyo, Japan) at 450-490 nm.

### TUNEL assay

TUNEL assay is used to label DNA fragmentation with fluorescein isothiocyanate (*In situ* Cell Death Detection Kit; Roche Molecular Biochemicals, Mannheim, Germany). Sections were incubated in TUNEL reaction mixture containing terminal deoxynucleotidyl transferase (TdT) for 60 min at 37°C. Sections were then observed and photographed under a fluorescence microscope (Olympus BX-51) with blue (450∼490 nm) excitation light. Negative controls were obtained by omission of the enzyme TdT.

### Immunohistochemistry staining

Immunohistochemical analyses were carried out as previously described [72]. After quenching of endogenous peroxidase activity and blocking of nonspecific binding with 10% normal goat serum, sections were incubated with rabbit anti-myeloperoxidase (MPO, a neutrophil marker; 1:1000; Dako, Carpinteria, CA, USA) or rabbit anti-Iba1 (a microglia/macrophage marker; 1:1000; Wako Pure Chemical Industries, Osaka, Japan) primary antibodies overnight. Further colorimetric detection was processed according to the instructions of a Vectastain Elite ABC Kit (Vector Laboratories, Burlingame, CA, USA) using diaminobenzidine as a peroxidase substrate. The specificity of the staining reaction was assessed in several control procedures, including omission of the primary antibody and substitution of the primary antibody with non-immune rabbit serum.

### Double immunofluorescence staining

To assess the cellular source of sEH, double immunofluorescence labeling was performed by simultaneous incubation of primary antibodies (anti-sEH, 1:1000; Santa Cruz Biotechnology, Santa Cruz, CA, USA) with mouse anti-neuronal nuclei antigen (NeuN, a neuronal marker; 1:100, Millipore, Billerica, MA, USA), rat anti-glial fibrillary acidic protein (GFAP, an astrocyte marker ; 1:200; Invitrogen, Camarillo, CA, USA), mouse anti-OX42 (a microglia/macrophage marker; 1:100; Wako), or rat anti-CD31(an endothelial cell marker; 1:100; BD Biosciences, CA, USA). Sections were incubated overnight at 4°C with anti-sEH antibody plus one of the antibodies to a specific cellular marker. To assess proinflammatory (M1) and anti-inflammatory (M2) microglia/macrophages, sections were incubated overnight at 4°C with rabbit anti-Iba1 (1:1,000; Wako), together with rat anti-CD16/32 (a classical M1 activation marker; 1:100; BD Biosciences), mouse anti-CD206 (an alternative M2 activation marker; 1:100; Bio-Rad Laboratories, Hercules, CA, USA) or rabbit anti-arginase 1 (an alternative M2 activation marker; 1:100; BD Biosciences). Sections were then washed, and incubated with Alexa Fluor 488- or Alexa Fluor 594-conjugated secondary antibodies (1:400; Molecular Probes, Eugene, OR) for 2 h. All sections were observed and photographed under a fluorescence microscope (Olympus BX-51). For primary microglial cultures, cells were plated onto poly-L-lysine coated cover slips in 24-well plate and were then incubated overnight with mouse anti-OX42 (1:100; Wako), together with rabbit-anti-iNOS (1:100; Chemicon), rabbit anti-p-P65, rabbit anti-p-P38, rabbit anti-p-JNK or rabbit anti-p-ERK p44/42 from Cell Signaling (Danvers, MA, USA). Subsequently the cells were incubated with Alexa Fluor 488- or Alexa Fluor 594-conjugated secondary antibodies (1:500; Molecular Probes, Eugene, OR) for 2 h and examined under a fluorescence microscope.

### Contusion volume and hemispheric enlargement assessment

Contusion volumes, residual tissue ratios, and hemispheric enlargement ratios were quantified using cresyl violet-stained sections at 20 rostral-caudal levels that were spaced 200 μm apart as previously described [69]. Sections were analyzed using ImageJ software (Version 1.50i, National Institutes of Health, Bethesda, MD, USA). The volume measurement was computed by summation of the areas multiplied by the interslice distance (200 μm). The preservation of cerebral tissue was evaluated by the ratio of the volume of the remaining ipsilateral cerebral hemisphere to the volume of the corresponding contralateral cerebral hemisphere. Brain edema was assessed by calculating the percentage of hemispheric enlargement using the following formula: ([ipsilateral hemisphere volume – contralateral hemisphere volume] / contralateral hemisphere volume) × 100%. Analysis was performed by two experimenters who were blinded to all animal groups. Inter-rater reliability was within 10%.

### Quantification of FJB, TUNEL, MPO and Iba1 staining

FJB, TUNEL, MPO and Iba1 stainings were quantified on three consecutive sections from the injury core at the level of 0.74 mm from the bregma. FJB-, TUNEL-, MPO-, and Iba1-positive cells were counted at a magnification of 200× in 3 randomly selected, non-overlapping fields with areas of 920 × 860 μm^2^. Iba1-positive resting microglia/macrophages were defined as resting if they contained relatively small cell bodies (<7.5 μm in diameter) with long slender processes [73]. Microglia were defined as activated when a cell body increased in size compared to resting microglia with short, thick processes and intense immunointensity. Activated microglia/macrophages were defined based on a combination of morphological criteria and a cell body diameter cutoff of 7.5μm. FJB-, MPO- and Iba1-positive cells were expressed as cells/field. Quantification of TUNEL staining was expressed as (TUNEL-stained nuclei/DAPI-stained nuclei) ×100%. Analysis was performed by two experimenters who were blinded to all animal groups. Inter-rater reliability was within 10%.

### Western blots

Western blots were performed as previously described [68]. A 4-mm coronal section from the injured area over the right parietal cortex was collected at 1 h, 3 h, 6 h, 12 h, 1 day, 4 days and 7 days following CCI or sham surgery. The liver sample was collected from the WT mouse as a positive control for the sEH antibody. BV2 cells were collected at 24h, N2A cells were collected at 48 h, and primary microglia were collected at 1h, 2h, 6h, and 24 h post-LPS or IFN-γ stimulation. All samples were centrifuged at 14,000×g for 30 min, and supernatants were used for further protein analysis. Protein concentration was determined by protein assay dye reagent at 595 nm. Protein samples were boiled with loading buffer at 100°C for 5 min, separated by electrophoresis on 8-12% sodium dodecyl sulfate-polyacrylamide gels, and transferred to Immobilon-P membranes (Millipore). Membranes were blocked with 5% milk in PBS-XT and probed with primary antibodies including rabbit anti-cleaved caspase-3 (1:1000), rabbit anti-p-P38 (1:1000), rabbit anti-P38 (1:2000), rabbit anti-p-ERK p44/42 (ERK p44/42; Thr202/Tyr204, 1:1000), rabbit anti-ERK (1:2000), rabbit anti-p-JNK (Thr183/Tyr185, 1:1000), rabbit anti-JNK (1:2000), rabbit anti-p-P65(1:1000) from Cell Signaling (Danvers, MA, USA), rabbit anti-ZO-1 (1:1000) and rabbit anti-claudin-5 (1:1000) from Invitrogen, mouse anti-iNOS (1:1000; Cayman), and mouse anti-β-actin (1:10000, sigma-Aldrich, St. Louis, MO, USA). The membranes were then incubated with horseradish peroxidase-linked anti-rabbit or anti-mouse secondary antibodies (1:5000, Santa Cruz) for 1 h at 4°C. Protein band intensities were quantified using ImageJ software and were normalized to the corresponding β-actin intensity.

### Gelatin gel zymography

Zymography was performed as previously described [72]. Briefly, protein samples were prepared similar to Western blot but without boiling. Samples were equally loaded and separated by 10% triglycine gel with 0.1% gelatin as the substrate. After separation, the gel was washed in distilled water, re-natured for 1 h with 2.5% Triton X-100 buffer at room temperature, and incubated for 48 h with developing buffer (0.05 M Tris-HCl pH 7.5, 0.2 mol/L NaCl, 5 mmol/L CaCl_2_, 0.05% Brij-35, 0.2 mmol/L NaN_3_) at 37°C. Then, the gel was stained with 0.05% Coomassie R-250 dye (Sigma-Aldrich) for 30 min and appropriately de-stained. Gelatinolytic activity (MMP-9, 97 kDa) was determined as showing clear bands at the appropriate molecular weights.

### Enzyme-linked immunosorbent assay

Protein samples were collected as in Western blot at day 1 and day 4 following CCI or sham surgery and in primary microglia cultures. IL-1β, IL-6, MIP-2, MCP-1 from R&D Systems (Minneapolis, MN, USA), EETs (MyBiosource, San Diego, CA, USA), and 14,15-DHET (Detroit R&D Inc., Detroit, MI, USA) were measured using ELISA kit. All samples and standards were assayed in duplicate according to the manufacturer’s instructions.

### NO production and cell viability

NO production was assessed by measuring the nitrite levels of the culture supernatants with Griess reagent (Sigma-Aldrich). Cell viability was assessed using MTT reduction assay (Sigma-Aldrich). The experiments were repeated four times with different batches of primary cultures.

### Statistical analyses

Data are presented as the mean and standard error of the mean (mean ± S.E.M). One-way or two-way analysis of variance (ANOVA) followed by post-hoc Bonferroni evaluation was used for multiple groups to determine significant differences on behavioral tasks, Western blots, brain water contents, BBB permeability, MMP-9 zymmography, ELISA and immunofluoresent stainings. Student’s t-test was used to test the difference between two groups on histology. Statistical significance was set at *P* < 0.05.

## SUPPLEMENTARY MATERIALS FIGURES


