# Supplementary material for: Deletion or inhibition of soluble epoxide hydrolase protects against brain damage and reduces microglia-mediated neuroinflammation in traumatic brain injury
Source: Oncotarget. 2017 Sep 21;8(61):103236–60. doi: 10.18632/oncotarget.21139 (PMC5732724; doi:10.18632/oncotarget.21139)
Supplement: Supplementary file 1 [file oncotarget-08-103236-s001.pdf]

Deletion or inhibition of soluble epoxide hydrolase protects against brain damage and reduces microglia-mediated neuroinflammation in traumatic brain injury

SUPPLEMENTARY MATERIALS

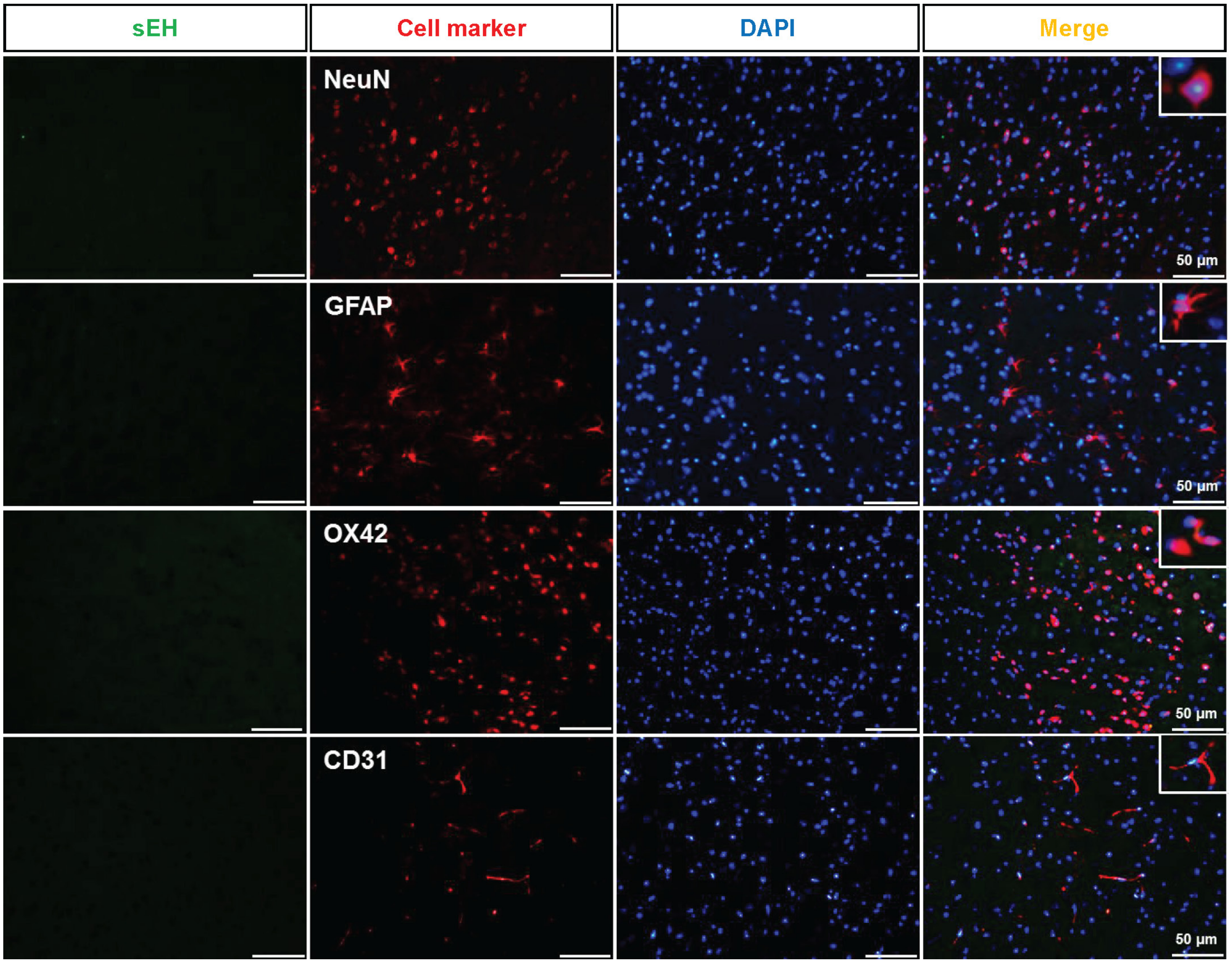

Supplementary Figure 1: Identification of sEH-positive cells 1 day post-TBI in the peri-contussional area of sEH KO mice by double immunofluorescent labeling. sEH immunoreactivity is shown in green and immunolabeling of cell markers is shown in red. sEH immunoreactivity was not detected in the ipsilateral hemisphere of sEH KO mice after CCI induction.

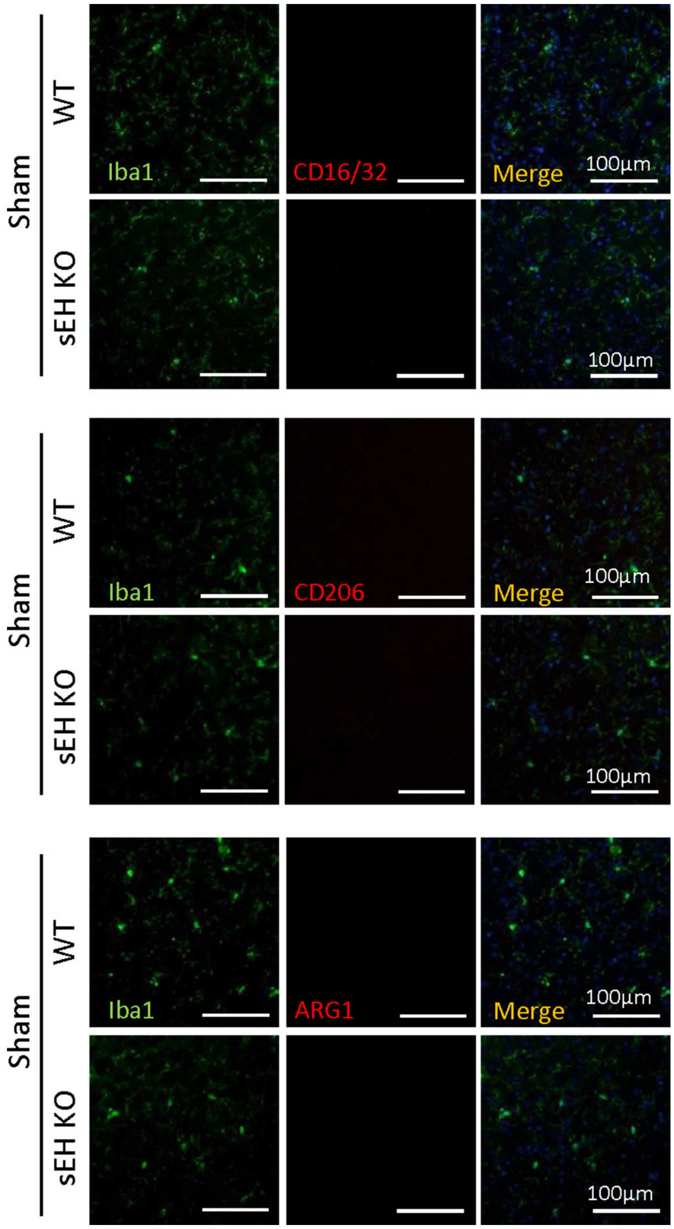

Supplementary Figure 2: Double-immunofluorescence of CD16/32, CD206 and arginase 1 of WT and sEH KO mice following sham surgery. Representative double-immunofluorescence of CD16/32 (classical activation marker; red), CD206 or arginase 1 (ARG1, alternative activation markers; red) and Iba1 (green) in the cortex (see the red box in the brain atlas coronal brain section in Figure 3C) of WT and sEH KO mice following sham surgery. The scale bar is 100 µm.

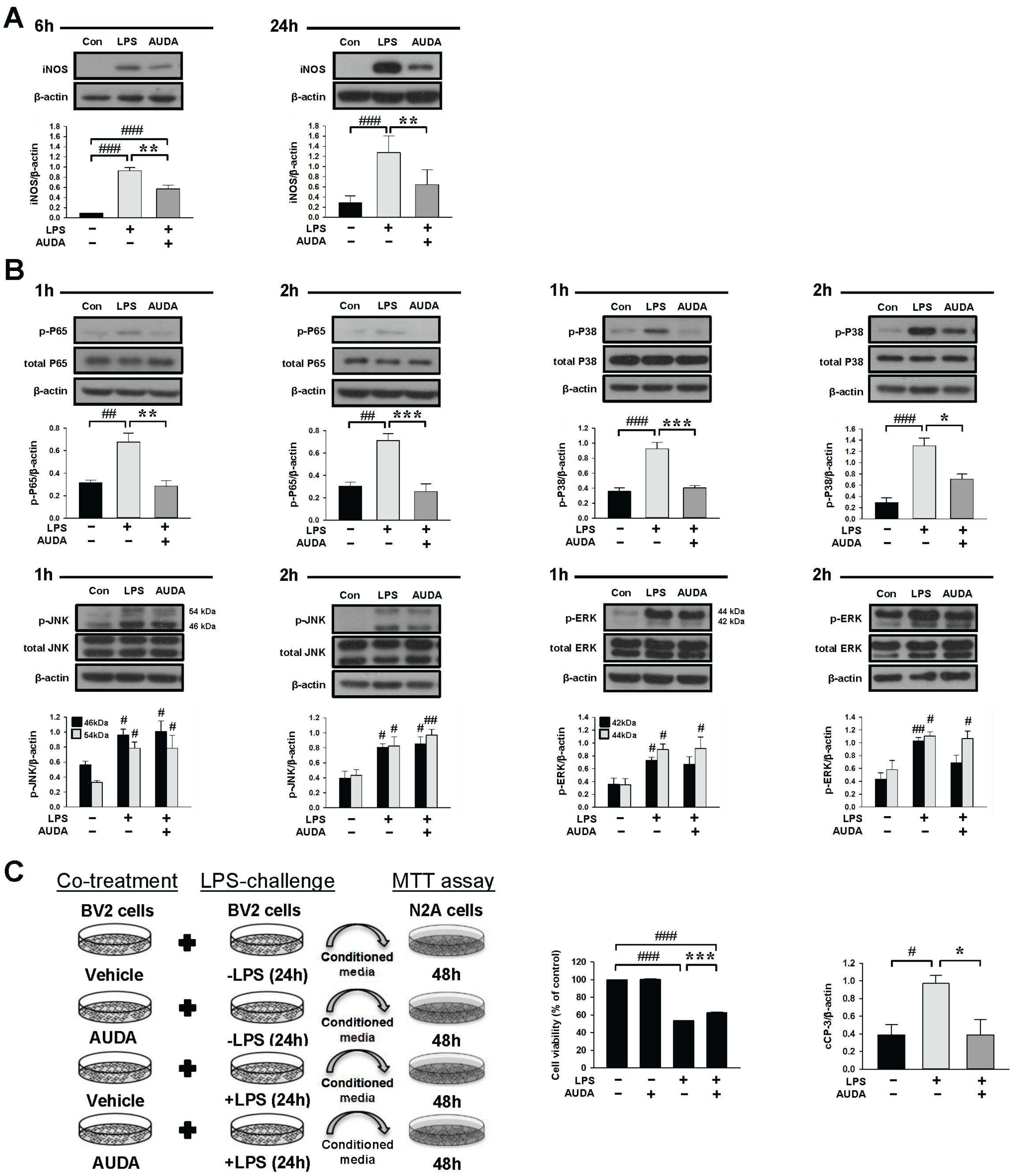

Supplementary Figure 3: Pharmacological inhibition of sEH inhibited LPS-induced P65 NF-κB and P38 MAPK activation in cultured microglia, and attenuated microglia-mediated neurotoxicity *in vitro*. **(A)** Representative immunoblots and bar graphs show that co-treatment of 10μM AUDA with LPS significantly reduced iNOS protein levels at 6 h and 24h. **(B)** Representative immunoblots and bar graphs show that co-treatment of 10μM AUDA with LPS significantly reduced LPS-induced P65 and P38 phosphorylation at 1 h and 2 h, but did not affect JNK or ERK phosphorylation in primary microglia. **(C)** Experimental scheme of neuronal survival in N2A cells in response to LPS-treated BV2-conditioned media with or without AUDA pretreatment. BV2 microglia were incubated with LPS in the absence (LPS-CM) or presence of 10μM AUDA (LPS/AUDA-CM) for 24 h. Cell-free supernatant fractions were applied to N2A cells for 48 h to evaluate the changes in cell viability and cleaved caspase-3 level. Neuronal cell death increased after exposure to LPS-treated conditioned microglial media; the effect was significantly reduced by microglia pretreatment with 10μM AUDA. Western blot analysis showed that AUDA significantly reduced the cleaved caspase-3 level compared with N2A cells treated with conditioned microglia media alone. cCP-3: cleaved caspase-3. Values are presented as mean ± S.E.M of four independent experiments. $^{\#}P < 0.05$, $^{\#\#}P < 0.01$, $^{\#\#\#}P < 0.001$ vs. normal control; $^{*}P < 0.05$, $^{**}P < 0.01$ vs. LPS stimulation alone (one-way ANOVA).
